# Protective effect of clusterin on rod photoreceptor in rat model of retinitis pigmentosa

**DOI:** 10.1371/journal.pone.0182389

**Published:** 2017-08-02

**Authors:** Andrew Vargas, Hwa Sun Kim, Erika Baral, Wan-Qing Yu, Cheryl Mae Craft, Eun-Jin Lee

**Affiliations:** 1 Mary D. Allen Laboratory for Vision Research, USC Roski Eye Institute, Department of Ophthalmology, Keck School of Medicine of the University of Southern California, Los Angeles, California, United States of America; 2 Department of Biological Structure, University of Washington, Seattle, Washington, United States of America; 3 Department of Cell & Neurobiology, Keck School of Medicine of the University of Southern California, Los Angeles, California, United States of America; 4 Neuroscience Graduate Program, University of Southern California, Los Angeles, California, United States of America; 5 Department of Biomedical Engineering, University of Southern California Viterbi School of Engineering, Los Angeles, California, United States of America; University of Florida, UNITED STATES

## Abstract

Retinitis Pigmentosa (RP) begins with the death of rod photoreceptors and is slowly followed by a gradual loss of cones and a rearrangement of the remaining retinal neurons. Clusterin is a chaperone protein that protects cells and is involved in various pathophysiological stresses, including retinal degeneration. Using a well-established transgenic rat model of RP (rhodopsin S334ter), we investigated the effects of clusterin on rod photoreceptor survival. To investigate the role of clusterin in S334ter-line3 retinas, Voronoi analysis and immunohistochemistry were used to evaluate the geometry of rod distribution. Additionally, immunoblot analysis, Bax activation, STAT3 and Akt phosphorylation were used to evaluate the pathway involved in rod cell protection. In this study, clusterin (10μg/ml) intravitreal treatment produced robust preservation of rod photoreceptors in S334ter-line3 retina. The mean number of rods in 1mm^2^ was significantly greater in clusterin injected RP retinas (postnatal (P) 30, P45, P60, & P75) than in age-matched saline injected RP retinas (P<0.01). Clusterin activated Akt, STAT3 and significantly reduced Bax activity; in addition to inducing phosphorylated STAT3 in Müller cells, which suggests it may indirectly acts on photoreceptors. Thus, clusterin treatment may interferes with mechanisms leading to rod death by suppressing cell death through activation of Akt and STAT3, followed by Bax suppression. Novel insights into the pathway of how clusterin promotes the rod cell survival suggest this treatment may be a potential therapeutic strategy to slow progression of vision loss in human RP.

## Introduction

Retinitis Pigmentosa (RP) is the cause of inherited blindness that results from gene mutations [1–6]. Regardless of the underlying genetic defect, the disease begins with the degeneration of rods followed by the degeneration of cones. This event eventually leads to a rewiring of the inner-retinal neurons [3, 4, 7–12]. Most studies involving retinal degeneration models have focused on naturally occurring models of RP [7, 9, 13–17]. These models are great tools for understanding the dynamics and mechanisms of retinal degeneration. However, a flaw of the naturally occurring RP models is that most express recessive forms of RP. The transgenic models of S334ter rat and P23H rat express a rhodopsin gene mutation that account for more than 25% of autosomal dominant human RP patients [18–20]. In the RP rhodopsin S334ter-line3 retina, rods die in” hot spots” or clusters [21]. These clusters of rod death appear to be embedded in a background of random cell death in the P14-P17 whole-mount retinas. Subsequently around P21, the rod cell deaths are propagated and expanded radially away from these clusters and form holes in the rod mosaic [21]. Some rod holes are visible until P45, and by P60, surviving rods are scattered in the whole-mount in the S334ter retina[22].

Recently we discovered that retinal ganglion cells are still functional at later stage of S334ter rats. The recordings of RGCs at P60 (i.e. nearly all rods were lost, but cones remained) showed that distinct RGC types are functionally intact. This result indicates that parallel processing of visual pathways remains largely intact in S334ter at P60 [23].

Clusterin, a multifunctional heterodimeric glycoprotein (70–80 kDa), is constitutively synthesized by a variety of tissues and plays a role in several biological events such as programmed cell death, lipid transport, sperm maturation, complement regulation and barrier cytoprotection [24–32]. Up-regulation of clusterin has been reported in response to pathological conditions, such as Alzheimer’s and Pick’s diseases[33–36], scrapie[37], spinal cord injury [38], axotomy [39], retinal ischemia [40], diabetic retinopathy [41] and retinal degeneration (RD) [42–46] including light-induced retinal degeneration [47–49] and RP [26, 50]. In spite of these various findings, the function of clusterin in these pathological conditions remains unknown. In the past, efforts to reduce cell death with an application of clusterin have produced promising results in various pathological conditions. For example, clusterin has been reported to be a cytoprotective protein in oxidative stress-induced cell death in the retinal pigment epithelial (RPE) cells [51, 52], hypoxia-induced injury [53], diabetic retinopathy [54, 55], and cerebral ischemic injury [56]. Furthermore, clusterin knockout mice have suggested that clusterin protects mice from the pathological consequences of inflammation [56, 57] and age-dependent deposition of antibody-containing aggregates in the kidney [58]. These experiments suggest that clusterin functions as the physiological defense to maintain cell viability and is involved in the suppression of pro-death signals after increased cellular injury.

In the present study, we investigated the neuroprotective feature of clusterin in a transgenic rat model of RP. We show for the first time that clusterin intravitreal treatment produced a robust preservation of rod photoreceptors in S334ter-line3 retina and Bax activity was significantly reduced. In addition, clusterin treatment induced Akt and STAT3 phosphorylation.

## Materials and methods

### Animals

The Veterinary Authority of University of Southern California’s Institutional Animal Care and Use Committee approved all procedures. The homozygous S334ter-line-3 (albino Sprague Dawley, SD), a transgenic RP prototype with a rhodopsin mutation caused by truncated murine opsin gene at Serine residue 334 (S334ter-line-3). Female rats were obtained from Matthew LaVail, Ph.D. (University of California San Francisco, CA) and were mated with Long-Evans (LE) male rats to generate heterozygous offspring. The heterozygous S334ter line 3 was designated as RP in this study. For controls, age-matched SD rats (Harlan, Indianapolis, IN) were used. Female or male SD rats and RP rats were euthanized at postnatal (P) days 15, 16, 17, 30, 45, 60, and P75 (number (n) = 9~12 and n = 6, respectively for each stage). For all experiments, animals were kept in cyclic 12-hour light/dark conditions with free access to food and water.

### Administration of clusterin

Clusterin (#2937-HS, R&D Systems, Minneapolis, MN) was prepared in phosphate buffered saline (PBS). For adjustment of clusterin dosage, 2 μl of three concentrations (10, 20 and 50 μg/ml in PBS) were administered by intravitreal injection with a Hamilton syringe (Sigma-Aldrich Corp) through the sclera on the temporal side of a P30 normal eye under anesthesia. Induction of anesthesia was done by intraperitoneal (IP) injection of ketamine (20 mg/kg; KETASET, Fort Dodge, IA, USA) and xylazine (5 mg/kg, X-Ject SA; Butler, Dublin, OH, USA) with similar procedures as our published protocols [22, 59, 60]. Following injections, veterinary ophthalmic antibacterial ointment was applied to prevent drying of cornea and infection. After preliminary testing of clusterin dosages, 10 μg/ml was selected throughout this current study. The developmental stage of RP for the intravitreal injection of clusterin or saline was postnatal days (P) 15; a peak period of rod photoreceptor cell death [11]. We divided three experimental groups: (1) RP animal injected with saline alone in both eyes, RP Saline; (2) RP animal injected with saline in right eye, RP saline (Rt), and (3) clusterin in left eye, RP Clusterin (Lt). The Saline (Rt) and Clusterin (Lt) are eyes from the same RP animals. For all experiments, clusterin and saline were injected at the same time of the day, Zeitgeber Time (ZT) 4 (ZT 0 defined as the moment lights were turned on), and tissues were isolated at each respective time point [61]. For all experiments, animals were kept in cyclic 12-hour light/dark conditions.

### Tissue preparation

Previous detailed protocols for tissue preparation were described [59, 60]. Briefly, animals were anesthetized by IP injection of Euthasol (40 mg/kg; Virbac Corporation, Fort Worth, TX) and the eyes were enucleated for collection of retinal tissue. Then, animals were euthanized by administration of an overdose of Euthasol. For secondary method we performed thoracotomy or decapitations. The lens and the anterior segment were removed and eyecups were fixed in 4% paraformaldehyde in 0.1 M phosphate buffer (PB), for 90 minutes at 4°C. Following fixation, the retinas were isolated from eye cups for whole mount staining and cryostat sectioning. For cryostat sectioning, eye cups were first transferred to 30% sucrose in PB overnight at 4°C, then were embedded in Optimal Cutting Temperature medium (OCT, Tissue-Tek, Elkhart, IN), frozen in liquid nitrogen and subsequently vertically sectioned on a Leica cryostat (Leica Biosystems Inc, Buffalo Grove, IL) at a thickness of 20μm.

### Immunohistochemistry

The detailed protocols for immunohistochemistry were performed as previously published [21, 60, 62]. Briefly, sections were incubated with 10% normal donkey serum (NDS) (#017-000-121, Jackson ImmunoResearch Laboratories, West Grove, Pennsylvania, dilution 1:10) for 1 hour at room temperature (RT), then incubated overnight with primary antibodies: goat polyclonal antibody directed against clusterin α (#sc-6420, Santa Cruz Biotechnology, Dallas, TX, dilution 1:1,000); rabbit polyclonal directed against glial fibrillary acidic protein (GFAP)(#G9269, Sigma-Aldrich Corp, St. Louis, MO, dilution 1:1,000); phosphorylated Signaling Transducer and Activator of Transcription 3 (pSTAT3)(#9145S, Cell Signaling Technologies, Danvers, MA, dilution 1:100); mouse monoclonal antibody against Glutamine Synthase (GS) (#MAB302, Millipore, Billerica, Massachusetts, dilution 1:10,000); mouse monoclonal antibody directed against rhodopsin (rho 1D4[63], dilution 1:1,000). Then samples were washed in PBS, and incubated for 2 hours at RT in corresponding secondary antibodies with carboxymethylindocyanine (Cy3)-conjugated affinity-purified donkey anti-rabbit IgG (Jackson ImmunoResearch Laboratories, dilution 1:500), Cy3-conjugated affinity-purified donkey anti-mouse IgG (Jackson ImmunoResearch Laboratories, dilution 1:500), Alexa 488-conjugated donkey anti-mouse IgG (Molecular Probes, Eugene, OR, dilution 1:300), or Alexa 488-conjugated donkey anti-goat IgG (Molecular Probes, Eugene, OR, dilution 1:300). Next, sections were washed three times for 10 minutes each with PB and cover-slipped with Vectashield mounting medium (Vector Labs, Burlingame, CA). Similar procedures to the ones described above were used for whole-mount immunofluorescence staining. Additionally, to enhance antibody penetration, tissues were treated with 1% Triton X-100 in PBS before blocking procedure with NDS, and primary antibodies were diluted in 0.5% Triton X-100 in PBS. For rhodopsin immunological staining in whole-mount retinas, the primary antibody incubation was for 2 days and the secondary antibody incubation was for 1 day. For double immunolabeling, vertical sections were incubated in a mixture of primary antibodies at 4°C for 24 hours, and then rinsed three times for 5 minutes each with PBS. Afterward, they were incubated with a mixture of corresponding secondary antibodies at RT for 2 hours. The images of sections and whole mounts were saved and processed with the Zeiss LSM-PC software under a Zeiss LSM 710 confocal microscope (Zeiss, NY), then were adjusted using Adobe Photoshop 7.0 (Adobe Systems, San Jose, CA). Photoshop adjustment was carried out equally across sections and whole mounts.

### Terminal deoxynucleotidyl transferase dUTP nick end labeling (TUNEL)

Cell death was tested using an In Situ Cell Detection kit (#11 684 795 910, Roche Diagnostics, Mannheim, Germany) in line with the manufacturer’s recommendations. The vertical sections were incubated with TUNEL reactive mixture for 90 minutes in 37°C. The sections were then washed with 0.1M PB for 30 minutes and cover-slipped with Vectashield mounting medium.

### Outer nuclear layer thickness measurement

For nuclear layer staining, we embedded the eyecups in OCT embedding medium (n = 5). Eyecups were sectioned along the vertical meridian on a cryostat at a thickness of 10 μm. TOPRO-3 (Invitrogen, Carlsbad, CA; T3605, dilution 1:1,000) was incubated for 10 minutes and washed for 30 minutes with 0.1 M PB and cover-slipped with Vectashield mounting medium. The Zeiss LSM image browser software was used to measure the thickness of ONL. The measurements were taken within 2 mm from the optic nerve.

### Voronoi analysis

Voronoi analysis was used to quantify the distribution of rod cells stained with rhodopsin. The detailed instructions for the Voronoi analysis were described in our previous study [60, 64]. Briefly, confocal micrographs of the retinas (n = 3–5 animals for each group) were taken at the mid-peripheral region (2 mm away from optic disc) of the superior temporal retina, covering a 1 x 1 mm^2^ area. At these locations we made serial optical sections using a confocal microscope. By following each rhodopsin-positive rod throughout the sections, we ensured that every rhodopsin positive cell in the selected region was counted. Using these images, the Voronoi domain of each cell was generated. The coefficient of clustering (CC) was also analyzed by the ratio between the global coefficient of variance and average local coefficient of variance in Voronoi domain sizes.

### Immunoblot analysis

Immunoblot analysis was performed on whole retinal cell lysate as previously reported [65, 66]. Briefly, each frozen retina was homogenized in ice-cold Radioimmunoprecipitation assay (RIPA) buffer, supplemented with EDTA-free proteinase inhibitor cocktail (Roche, Basel, Switzerland). After homogenization, samples were centrifuged at 16,000 g for 10 min and supernatant was collected to measure protein concentrations using Pierce BCA assay kit (Thermo Scientific, Rockford, IL). 45 μg of protein per retina were applied to electrophorese on a 10 ~ 12% sodium dodecyl sulfate-polyacrylamide gel (SDS-PAGE), and then were transferred to nitrocellulose membranes (LI-COR Biotechnology, Lincoln, NE). After 1 hour of blocking with Odyssey blocking buffer (LI-COR Biotechnology), membranes were incubated overnight with antibodies for anti β-actin (#A5441, Sigma, dilution 1:5000) and either anti-clusterin α (Santa Cruz, dilution 1:500), anti-pAkt (#4060S, Cell Signaling, dilution 1:500), anti-Akt (#4691S, Cell Signaling, dilution 1:500), anti-STAT3 (#12640S, Cell Signaling, dilution 1:500), anti-pSTAT3 (Cell Signaling, dilution 1:500), anti-Bax (#14796S, Cell Signaling, dilution 1:500) or anti-caspase-3 (#9664S, Cell Signaling, dilution 1:500). For detection of clusterin by immunoblot analysis, we used anti-clusterin α to observe not only the alpha subunit as a part of mature form of clusterin, but also the clusterin precursor (~70 kDa, single polypeptide without disulfide links between alpha chain (36–39 kDa) and beta chain (34–36 kDa) [32]. Afterward, appropriate secondary antibodies conjugated to a fluorophore (680 nm or 800 nm) were used for detection using an infrared detection system (GENESys, Syngene, Frederick, MD). For all optical density analysis, we used National Institute of Health (NIH) Image J software version 1.50i to quantify the intensity of each band. Beta-actin was used as a loading control. Relative amounts of clusterin, pAkt, pSTAT3, caspase-3, and Bax were calculated by dividing the immunological intensity of clusterin, pAkt, pSTAT3, caspase-3, and Bax protein bands by the intensity of the β-actin protein. The average of the normal or saline-treated RP at 5 min was set as 100%.

### Statistical analysis

All the statistics were expressed as mean ± standard error of the mean (SEM). Student’s t-test was used for comparison (GraphPad Prism 6, La Jolla, CA). One-way ANOVA, two-way ANOVA and Fisher's least significant difference procedure (LSD test) were used to examine the differences among the group of means. The tests were performed by MATLAB version 8.2.0 (The MathWorks Inc., Natick, MA, USA) and graphs were generated by GraphPad Prism 6. The difference between the means of separate experimental groups was considered statistically significant at P < 0.05.

## Results

### Expression of clusterin in S334ter-line3 RP retina

**Fig 1:**
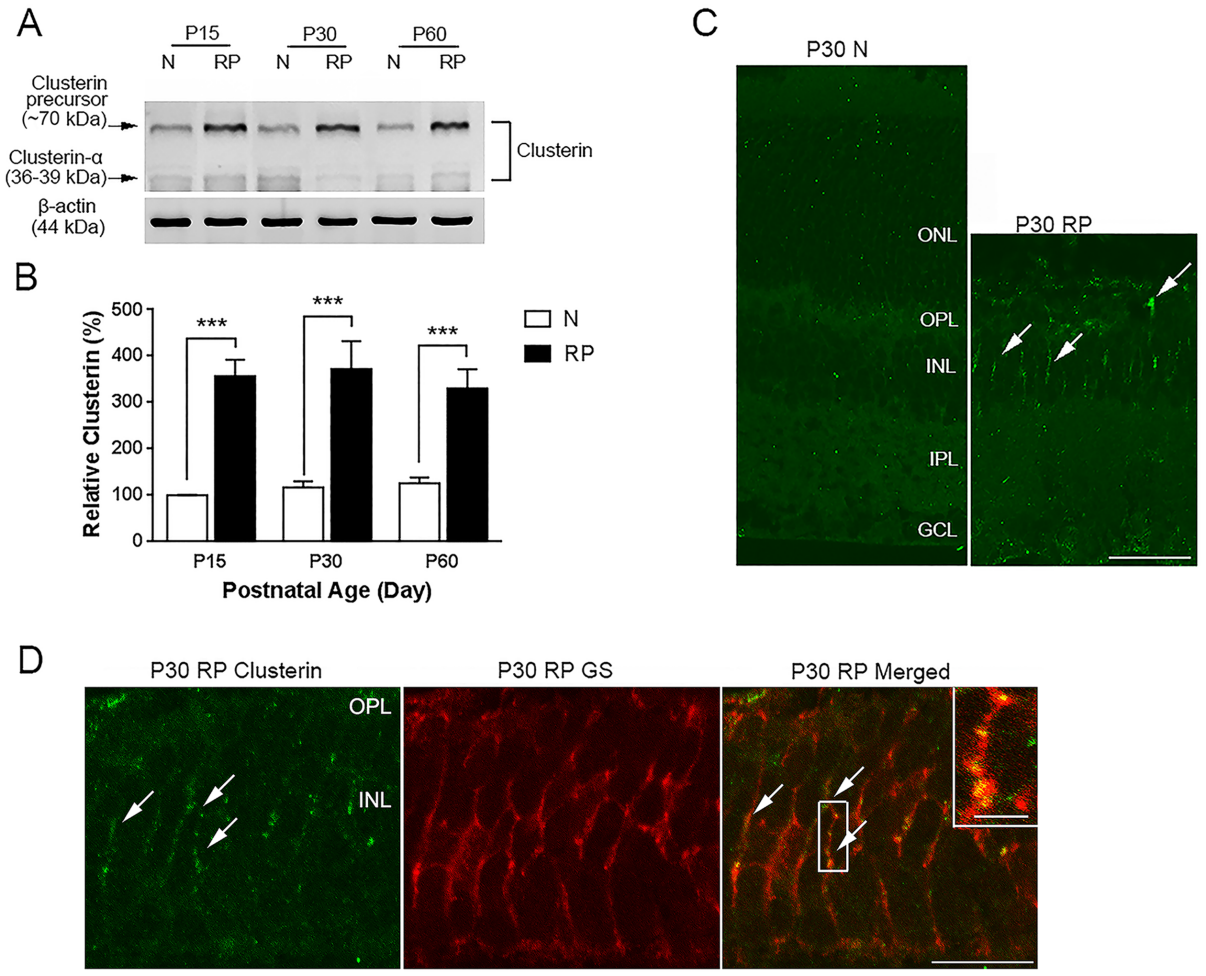
Expression of clusterin in normal and RP retinas. Immunoblot analysis showed clusterin-immunoreactive bands at ~70 kDa and ~36 kDa at P15, P30, and P60 normal and RP retinas (A). The clusterin precursor-immunoreactive band of ~70 kDa was significantly upregulated in retinal extracts of P15, P30, and P60 RP compared with retinal extracts from age-matched normal rat. Inconsistent expression of the precursor and clusterin-α was observed in normal and RP retinas. Densitometry analysis of immunoblots for clusterin precursor proteins was generated in histogram (B). Clusterin immunoreactivity was localized on vertical sections of P30 normal and P30 RP retinas (C). No clusterin immunoreactivity was detected in normal retina. In contrast, clusterin expression was observed in some processes in the INL of RP retina (arrows). Vertical sections labeled with anti-clusterin (green) and anti-glutamate synthase (GS, red) in P30 RP retinas (D). All clusterin immunoreactive processes in the INL were associated with processes of Müller cells (inset). ONL, outer nuclear layer; OPL, outer plexiform layer; INL, inner nuclear layer; IPL, inner plexiform layer; GCL, ganglion cell layer; P, postnatal; N, normal; RP, Retinitis Pigmentosa. Data represents mean ± SEM; *** P<0.0001. Scale bar = 50 μm, 10 μm in inset.

Increased clusterin expression is associated with retinal degenerative diseases [42–46] including light-induced RD [47, 48] and RP [50, 67]. To determine whether clusterin was up-regulated in our RP rat model, we first investigated the expression levels of clusterin in retinal extracts by immunoblot analysis using antibodies against clusterin-α. A clusterin-immunoreactive band was detected at ~70 kDa (clusterin precursor) and 36–39 kDa (clusterin-α). The result was consistent with previous studies showing multiple bands in a study when clusterin was first cloned, in human blood and cerebrospinal fluid (CSF) of patients with Alzheimer’s disease, and in colon cancer cells *in vitro* [68–70]. The clusterin precursor-immunoreactive band of ~70 kDa (Fig 1A) was present in normal and RP retinal extracts of P15, P30, and P60. However, the intensity of the clusterin precursor-immunoreactive band was stronger in RP retinas than in normal retinas. In contrast, the level of clusterin-α was relatively unchanged among the experimental groups (S1 Fig, S2 Table). Relative clusterin precursor expression was observed by measuring the optical density of clusterin-immunoreactive proteins in these retinas (Fig 1B). The clusterin precursor levels were ~3.5 times higher (*** P<0.0001) in RP retinas than in normal retinas. Our data indicated that clusterin expression was significantly elevated in RP retinas.

In P30 normal retina, clusterin immunohistochemical labeling was not observed. In contrast, clusterin expression was noted in some processes in the inner nuclear layer (INL) of RP retina (Fig 1C). To identify the immunoreactive profiles in the INL of RP, double immunohistochemical labeling experiments using antisera against the clusterin and glutamine synthase (GS, the Müller cell marker) were performed [71, 72]. We found that clusterin immunoreactive profiles co-localized with GS immunoreactivity (Fig 1D). These results verified that clusterin was present in processes of Müller cells in RP retina.

### Clusterin treatment affects rod photoreceptor cell survival in RP retina

**Fig 2:**
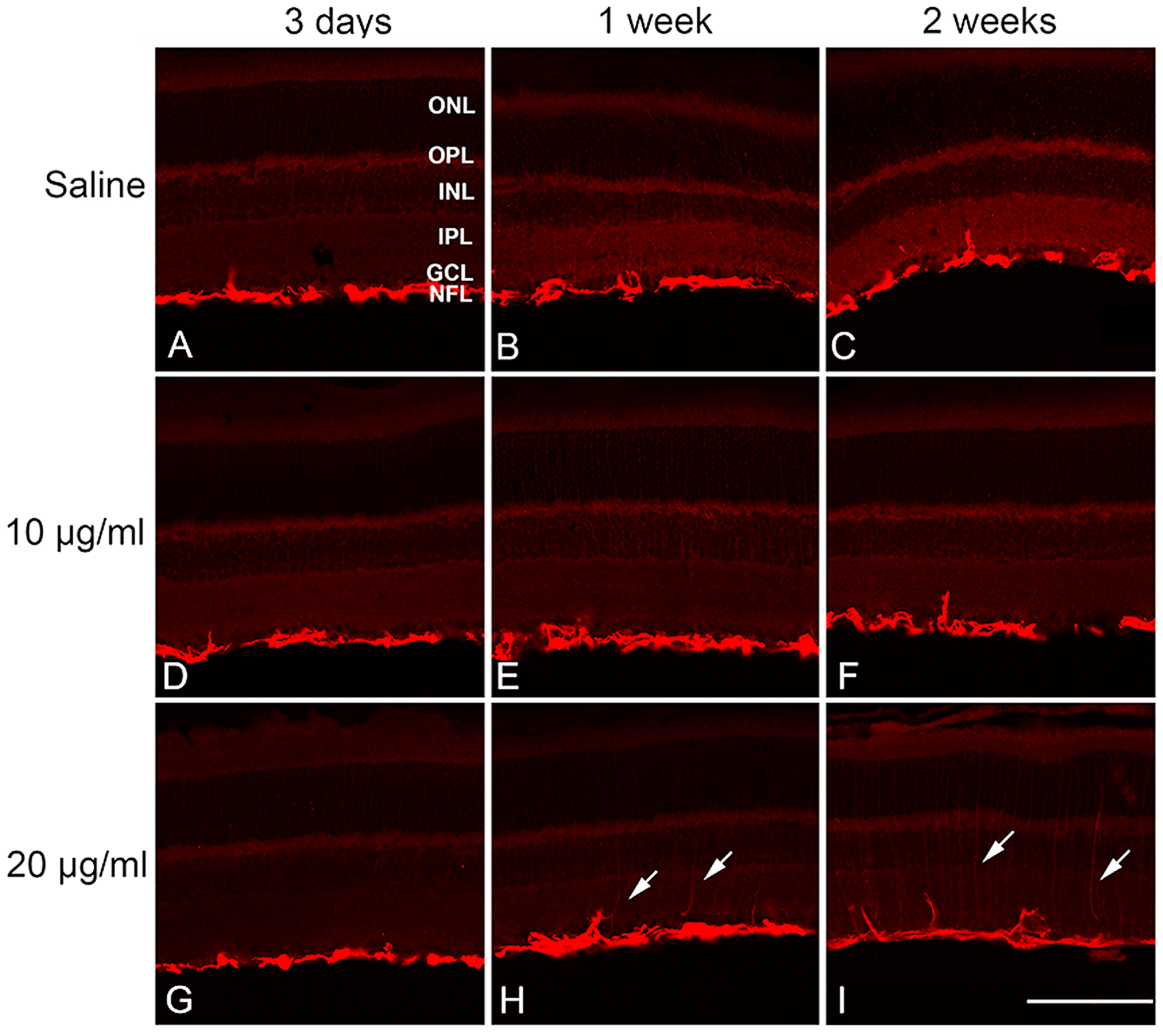
Gliosis was induced by clusterin at higher dose. Immunoreactivity of GFAP was localized on vertical sections of normal retinas. GFAP immunoreactivity was localized in the NFL after 3 days (A, D), 1 week (B, E), and 2 weeks (C, F) of saline and 10μg/ml clusterin post-injection. Moderate gliosis was seen at the dosage of 20μg/ml clusterin (H, I) at the NFL and inner retina. GFAP, glial fibrillary acidic protein; ONL, outer nuclear layer; OPL, outer plexiform layer; INL, inner nuclear layer; IPL, inner plexiform layer; GCL, ganglion cell layer. Scale bar = 100 μm.

The induction of clusterin expression in pathological conditions is still controversial since some reports suggest that clusterin is involved in apoptosis, whereas other reports demonstrated that clusterin participates in a protective role. Thus, we investigated if an exogenous application of clusterin could affect the rod survival in RP retina, with the hypothesis that clusterin was an “innate defender.” Before application of clusterin in RP, we tested three dosages of clusterin in normal retinas. Either saline or 10, 20, and 50 μg/ml of clusterin was injected and we observed the effects after 3 days (Fig 2A, 2D and 2G), 1 week (Fig 2B, 2E and 2H), and 2 weeks (Fig 2C, 2F and 2I). There was no detectable gliosis (Fig 2A–2F) or TUNEL positive cells (data not shown) in normal retina throughout the experimental periods except at the dosage of 20 μg/ml of clusterin where moderate gliosis was detected (Fig 2H and 2I, arrows). We also observed moderate gliosis and no cell death in 50 μg/ml injected retina (data not shown). Thus, we used 10μg/ml of clusterin as an appropriate dose to determine its influence on the RP retina.

**Fig 3:**
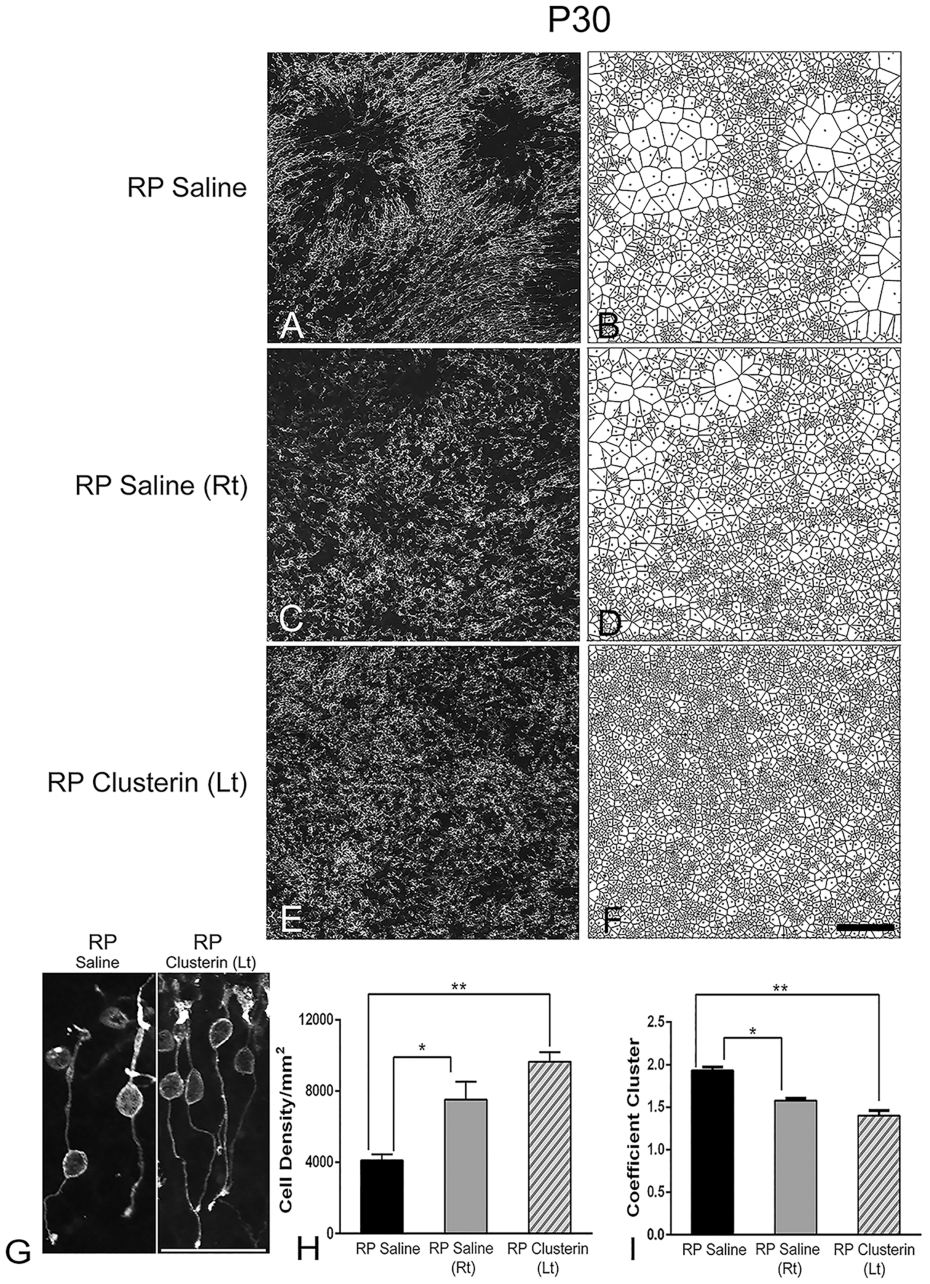
Clusterin delayed rod cell death and had bilateral effect. Confocal micrographs of whole-mounted retina labeled with rhodopsin at P30. One group of RP animals was treated with saline alone (A) and another group of RP animals was treated with saline in their right eye (C) and clusterin in their left eye (E) at P15. Voronoi domains are shown for distribution of rod cells (B, D, F). Rhodopsin immunoreactivity was in the segments, cell bodies, axon processes, and axon terminal of rods in whole mount RP Saline and RP Clusterin (Lt) retinas (G). The outer segments were shortened and distorted. The length of axon processes was varied among the rods. The overall morphology of rods was aquiline/curved. Rod cell number per 1 mm^2^ in clusterin treated left eyes was significantly higher than among the groups (H). Coefficient of clustering (CC) was significantly lower in both RP Saline (Rt) and RP clusterin (Lt) treated retinas compared to RP saline. Data presented as mean with SEM. *P = 0.01, **P = 0.001. Scale bar = 100 μm.

Intravitreal injection of clusterin (10 μg/ml) at P15, when rod photoreceptor cells show peak cell death in RP [11], resulted in significant protection of rod photoreceptors (Fig 3H). Additionally, we discovered that clusterin has a bilateral effect to rod cell survival and rod cell distribution as shown in Fig 3C–3F, 3H and 3I. For quantitative analysis, we divided the three experimental groups: (1) RP animal injected with saline alone, RP Saline; (2) RP animal injected with saline in right eye, RP saline (Rt), and (3) clusterin in left eye, RP Clusterin (Lt). We performed rho 1D4 (rhodopsin) immunohistochemical staining on these retinas and counted the rhodopsin positive cells from the individual optical sections that made up the reconstructed stack from the whole-mounts. The S334ter rhodopsin mutant removes both the trafficking signal to the outer segment and the rho 1D4 monoclonal antibody binding motif. The antibody rho 1D4 recognizes residues in the carboxyl-terminus of rhodopsin and thus it will detect only the endogenous rat rhodopsin and not the mutant rhodopsin, which lack the well-established role of the C-terminal trafficking motif [73].

In Fig 3A, 3C and 3E, we showed an example of a whole-mount retinas processed for rhodopsin immunohistochemical staining at P30 taken at the central part of the superior and temporal region of RP Saline (Fig 3A), RP Saline (Rt) (Fig 3C) and RP Clusterin (Lt) (Fig 3E) retinas. In RP retina, rhodopsin immunoreactivity was in the segments, cell bodies, axon processes, and axon terminal of rods in RP Saline, RP Clusterin (Lt) (Fig 3G), and RP Saline (Rt, data not shown). The outer segments were shortened and distorted. The length of axon processes was varied among the rods. The overall morphology of rods was aquiline/curved [21]. The mislocalization of rhodopsin or opsins in RP was consistent with previous studies [74–78] Thus, for quantitative analysis of rods, we counted the rhodopsin positive cell bodies from the individual optical sections that made up the reconstructed stack from the whole-mounts. In P30 RP Saline retinas, we observed holes in the rod mosaic (Fig 3A) [21]. In contrast, rod cells in P30 RP Saline (Rt) and RP Clusterin (Lt) retinas were more homogenously distributed (Fig 3C and 3E). The distribution of rods was quantified using Voronoi analysis (Fig 3B, 3D and 3F). We observed groups of large domains and small domains in RP Saline retina (Fig 3B). In contrast, RP Saline (Rt) and RP Clusterin (Lt) showed mixtures of large and small domains (Fig 3D and 3F). The summary graph demonstrated the mean rod density (Fig 3H) measured from the 1x1 mm^2^ sampling areas (for details, see Materials and methods) of RP Saline, RP Saline (Rt), and RP Clusterin (Lt). The mean rod cell density in RP Saline retinas at P30 was 4,104±343 cells/mm^2^. The density of rod cells changed with clusterin-treated RP. The density from the RP Saline (Rt) and RP Clusterin (Lt) retinas showed higher numbers of 7,529±994 and 9,650±534 cells/mm^2^ at P30, respectively. The one-way ANOVA analysis showed significant differences in mean among the different groups (Fig 3H, RP Saline vs. RP Saline (Rt) * P<0.01; RP Saline vs. RP Clusterin (Lt) ** P<0.001). There was no significant difference in the mean between RP Saline (Rt) and RP Clusterin (Lt). These data clearly demonstrated that clusterin treatment delayed rod death in RP retina. We also examined the impact on the outer nuclear layer (ONL) thickness in the different comparisons (i.e. RP saline vs. RP Clusterin (Lt); S2 Fig). Intravitreal injection of clusterin at P15 resulted in significant protection of ONL when examined at P21 and P30. The overall appearance of the ONL of RP Saline was thinner than the RP Clusterin (Lt) until P30. Measurement of the ONL thickness showed that the thickness in the RP clusterin retinas (P21, 14.5 ± 0.3 μm; P30, 12 ± 0.3 μm) is significantly greater (P21, P  =  0.0006; P30, P  =  0.0001) than in RP Saline retinas (P21, 13.2 ± 0.2 μm; P30, 10 ± 0.2 μm). At P45 the appearance of the ONL of RP Saline (4.2 ± 0.15 μm) is similar to RP Clusterin (Lt) (4.3 ± 0.1 μm), differing slightly in the number of cells in ONL. Thus, to examine the cell survival in the outer retina. we also counted the rhodopsin immunoreactive positive cell bodies from the whole-mounts and demonstrated the difference in mean rod density (Fig 3H) of RP Saline and RP Clusterin (Lt) retina. Next, we quantified the correlation between the sizes of neighboring domains by calculating the coefficient of clustering (CC). The details of the methodology of the coefficient of clustering were published [60]; however, briefly, the CC is the ratio of the global coefficient of variation to the average local coefficient of variation in Voronoi domain sizes. If a random distribution of Voronoi domain were shown, the CC would be close to 1. In contrast, small domains were neighboring other small domains and the large domains were close to each other, the CC would be greater than 1. The CC was high in RP Saline (1.93 ± 0.04) and became significantly lower with RP Saline (Rt) (1.57 ± 0.03) (*p = 0.0174) and RP Clusterin (Lt) (1.40 ± 0.06) (**p = 0.0011) (Fig 3I). Therefore, the rods were distributed more homogeneously in clusterin treated animals (i.e. RP Saline (Rt) and RP Clusterin (Lt)).

**Fig 4:**
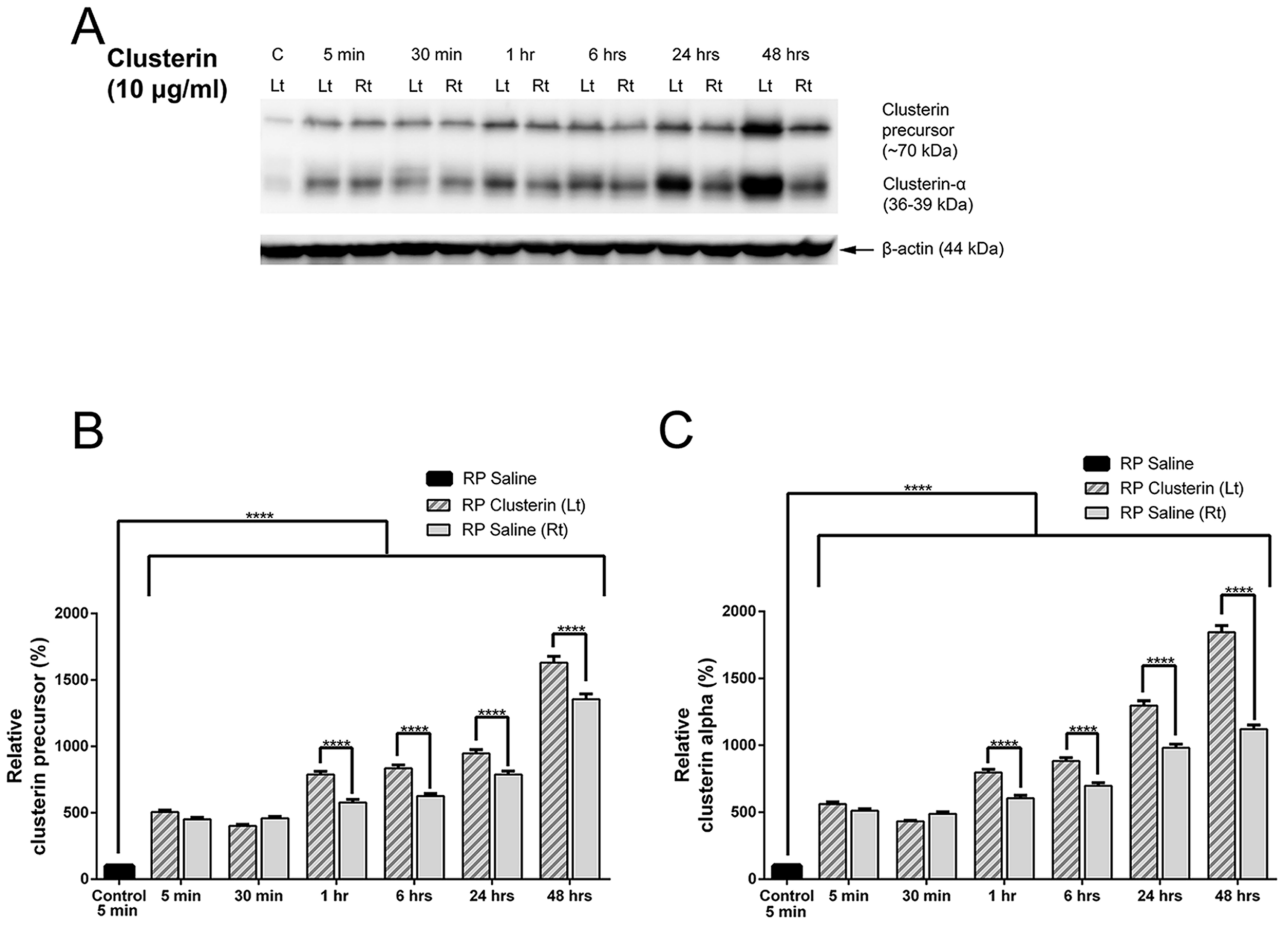
Clusterin expression was examined by immunoblot analysis in RP Saline, RP Saline (Rt), and RP Clusterin (Lt) retinas. Clusterin or saline was injected at P15 of RP, and then the retinas were collected at 5 minutes, 30 minutes, 1 hour, 6 hours, 24 hours, and 48 hours. A significant increase in clusterin (i.e. clusterin precursor and clusterin-α) was detected as early as 5 minutes in both RP Clusterin (Lt) and RP Saline (Rt) compared to that of 5minutes RP Saline retinas. The expression of clusterin did not change among the RP saline groups (i.e. 5 minutes, 30 minutes, 1 hour, 6 hours, 24 hours, and 48 hours, data not shown). Furthermore, the expression of clusterin was higher in the RP Clusterin (Lt) retinas than in the RP Saline (Rt) at 1hour after clusterin injection (A). Densitometry analysis of clusterin precursor (B) and clusterin alpha subunit (C) was shown by measuring the intensity relative to control β-actin (B, C). Data represents mean ± SEM, **** P<0.0001.

The bilateral effects on the other eye of clusterin injected animals were also detected by immunoblot analysis (Fig 4). Clusterin expression was examined by immunoblot analysis in RP Saline, RP Saline (Rt), and RP Clusterin (Lt) retinas. Clusterin or saline was injected at P15 of RP, and then the retinas were collected at 5 minutes, 30 minutes, 1 hour, 6 hours, 24hours, and 48 hours. The expression of clusterin (i.e. 5 minutes) did not change among the RP saline groups (i.e. 5 minutes, 30 minutes, 1 hour, 6 hours, 24 hours, and 48 hours, data not shown). In contrast, a significant increase in clusterin expression (i.e. clusterin precursor and clusterin-α) was detected as early as 5 minutes in both RP Clusterin (Lt) and RP Saline (Rt) compared to that of 5 minutes RP saline retinas. The expression of clusterin continues to increase until 48 hours in both Clusterin (Lt) and RP Saline (Rt). Furthermore, the expression of clusterin was higher in the RP Clusterin (Lt) retinas than in the RP Saline (Rt) at 1 hour after clusterin injection (Fig 4A). Densitometric analysis of clusterin precursor (Fig 4B) and clusterin alpha subunit (Fig 4C) was shown by measuring the intensity relative to the control, β-actin. The two-way ANOVA analysis showed significant differences in mean among the different groups, **** P<0.0001). Our results showed that more endogenous precursor of clusterin was produced after clusterin injection and suggested that the application of clusterin may have facilitated kinetically to the cleavage pathway. Furthermore, clusterin levels changed after clusterin injection and the injection of clusterin on the left eye affected the right eye of the same animal, suggesting that there is a bilateral effect on the other eye of clusterin injected animals.

**Fig 5:**
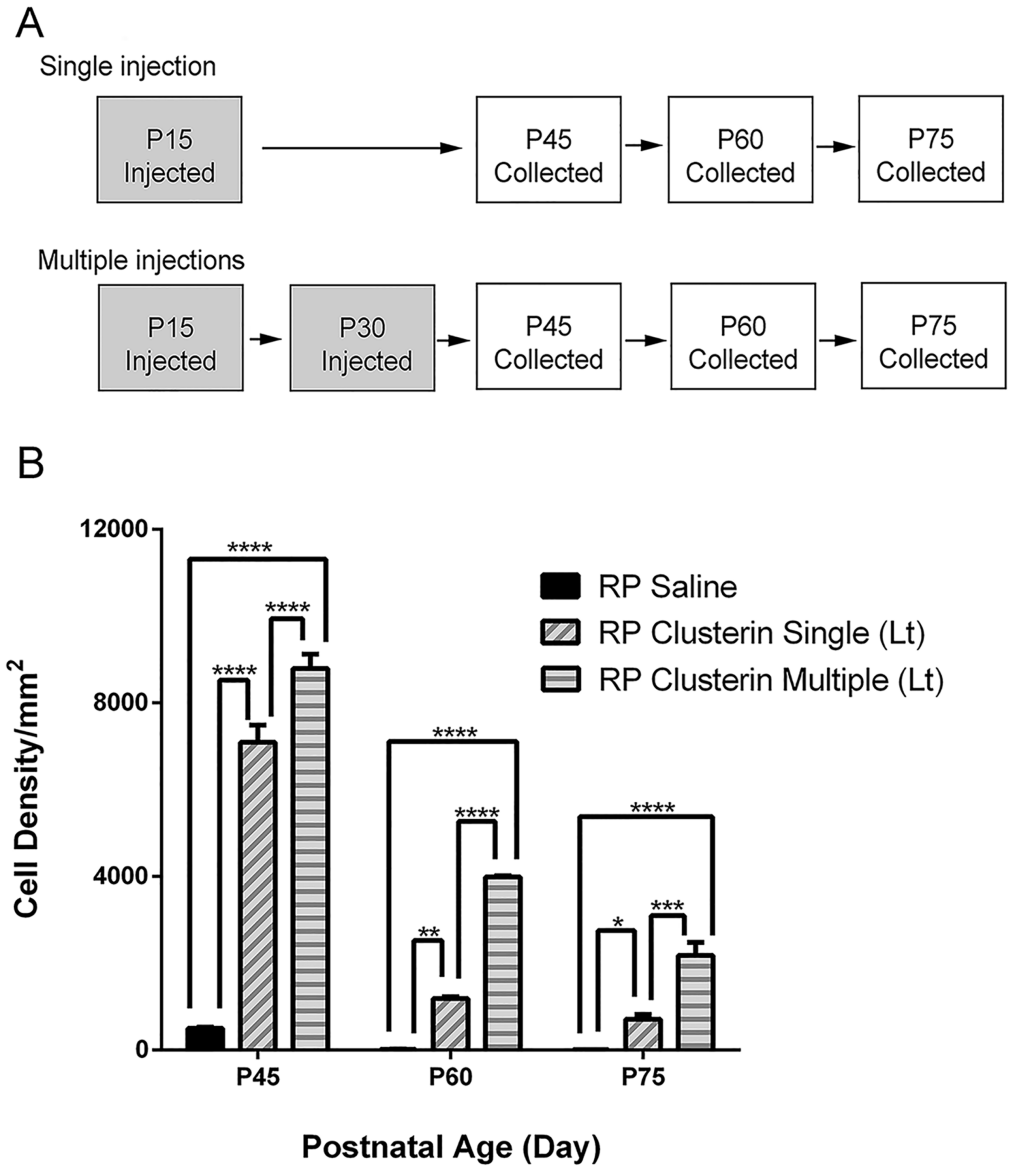
Clusterin delays rod death. For single injection, saline or clusterin were injected at P15 and retinas was collected at P45, P60, and P75. For multiple injections, saline or clusterin was injected at P15 and P30, and retinas were collected at P45, P60, and P75 (A). Rod cell number per 1 mm^2^ was significantly higher in clusterin treated retinas compared to saline treated retinas at all ages (B). Multiple treatments of clusterin improved rod survival better than that of single treatment of clusterin. Data represents mean ± SEM, *P = 0.02, **P = 0.002, ***P = 0.0002, ****P < 0.0001. P, postnatal. Scale bar = 100μm.

We further investigated the impact of clusterin treatment on rod survival in a more advanced stage of RP (Fig 5). We first performed a single injection at P15 and collected retinas at P45, P60, and P75 (i.e. RP Clusterin Single (Lt)). For multiple injections, we injected clusterin at P15 and P30 and collected retinas at P45, P60, and P75 (i.e. RP Clusterin Multiple (Lt), Fig 5A). Since clusterin showed some bilateral effects on the other eye of clusterin injected animal (Figs 3 and 4), from now on, we compared the RP animals between RP saline (RP animal injected with saline alone) and RP Clusterin (Lt) (RP animal injected with clusterin in left eye). We examined the rod photoreceptor cell density after collecting the retinas at P45, P60, and P75, following single or multiple injections and compared the cell number with each group of RP Saline retinas, P45, P60, and P75. Quantitative analysis was shown by counting rhodopsin immunohistochemically stained cells taken at the central part of RP Saline, RP Clusterin Single (Lt), and RP Clusterin Multiple (Lt) (Fig 5B) retinas at P45, P60, and P75. The cell density graph illustrates the mean rod cell density (Fig 5B) measured from the 1x1 mm^2^ sampling areas of RP Saline, RP Clusterin Single (Lt), and RP Clusterin Multiple (Lt) retinas. The mean density of cells in saline—treated RP retinas at P45, P60, and P75 were 500±24/mm^2^, 18±2/mm^2^, and 7±1/mm^2^, respectively. The density from the clusterin—single treated RP retinas at P45, P60, and P75 were 7,094±391/mm^2^, 1,188±36/mm^2^, and 711±111/mm^2^, respectively. The density from the clusterin—multiple treated RP retinas showed higher number of 8,790±337/mm^2^, 3,988±31/mm^2^, and 2,181±298/mm^2^, respectively. The two-way ANOVA analysis showed significant differences between the mean of different groups of retinas and the different postnatal days (Fig 5B, *P = 0.02, **P = 0.002, ***P = 0.0002, ****P < 0.0001).). These results indicated that clusterin treatment effectively delayed rod photoreceptor cell death in RP. Furthermore, multiple treatments of clusterin improved rod survival better than that of single treatment of clusterin.

### Clusterin activates Akt phosphorylation in retina of RP

**Fig 6:**
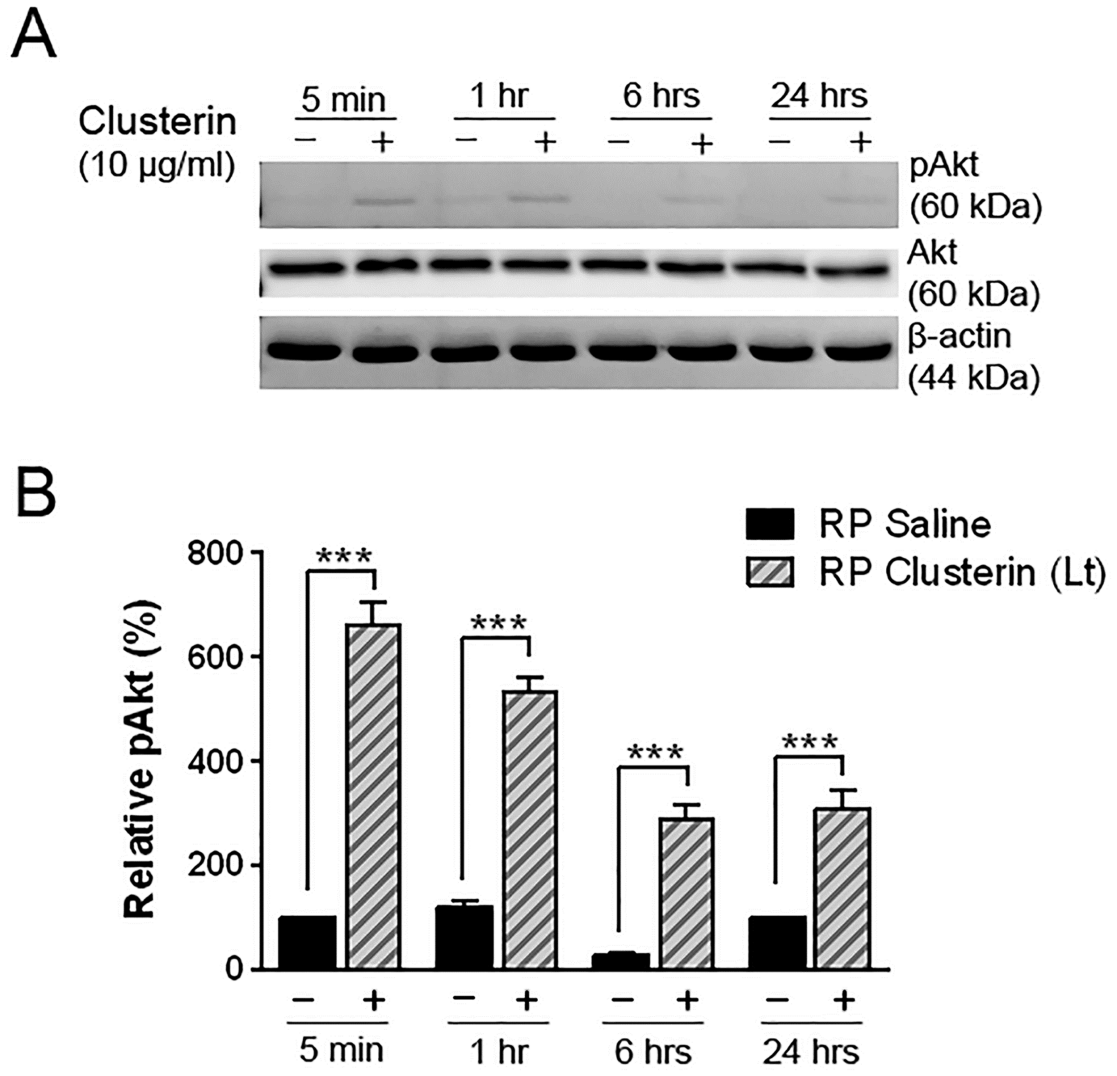
Induction of pAkt by clusterin in RP retina. Immunoblot analysis of phosphorylated Akt (60kDa) was examined in saline- and clusterin-treated RP retinas (A). Retinas were collected at 5 min, 1 hour, 6 hours, and 24 hours after injection at P15. Activation of Akt was detected as early as 5 min after clusterin injection. The saline-treated retinas (-) showed lower activation of Akt at different time points, compared to the clusterin-treated retinas (+). Densitometry analysis of immunoblots for phosphorylation of Akt by clusterin was generated in histogram (B). Beta-actin served as the loading control to gain relative pAkt activation value. Data represents mean ± SEM, *** P<0.0001. P, postnatal. Akt, Protein kinase B; pAkt, Phosphorylated Protein kinase B.

Studies have shown that activation of Akt by clusterin protects human RPE cells and cardiomyocytes from the oxidative stress, and mesenchymal stem cells against ischemic injury [51, 53, 79]. In the present study, we also determined whether clusterin induces Akt phosphorylation in clusterin-treated retinas or not. Clusterin was applied at P15 of RP, and then the retinas were harvested at 5 minutes, 1 hour, 6 hours, and 24 hours. A significant increase in phosphorylation of Akt was detected as early as 5 minutes after clusterin injection (Fig 6A and 6B). In contrast, the group of saline-treated retina showed weaker expression of pAkt at different time points, compared to clusterin-treated group (***P<0.0001). Beta-actin and Akt served as the loading control. These results supported that clusterin acts via Akt activation to play a role in protection in RP retina.

### Clusterin activates STAT3 phosphorylation

**Fig 7:**
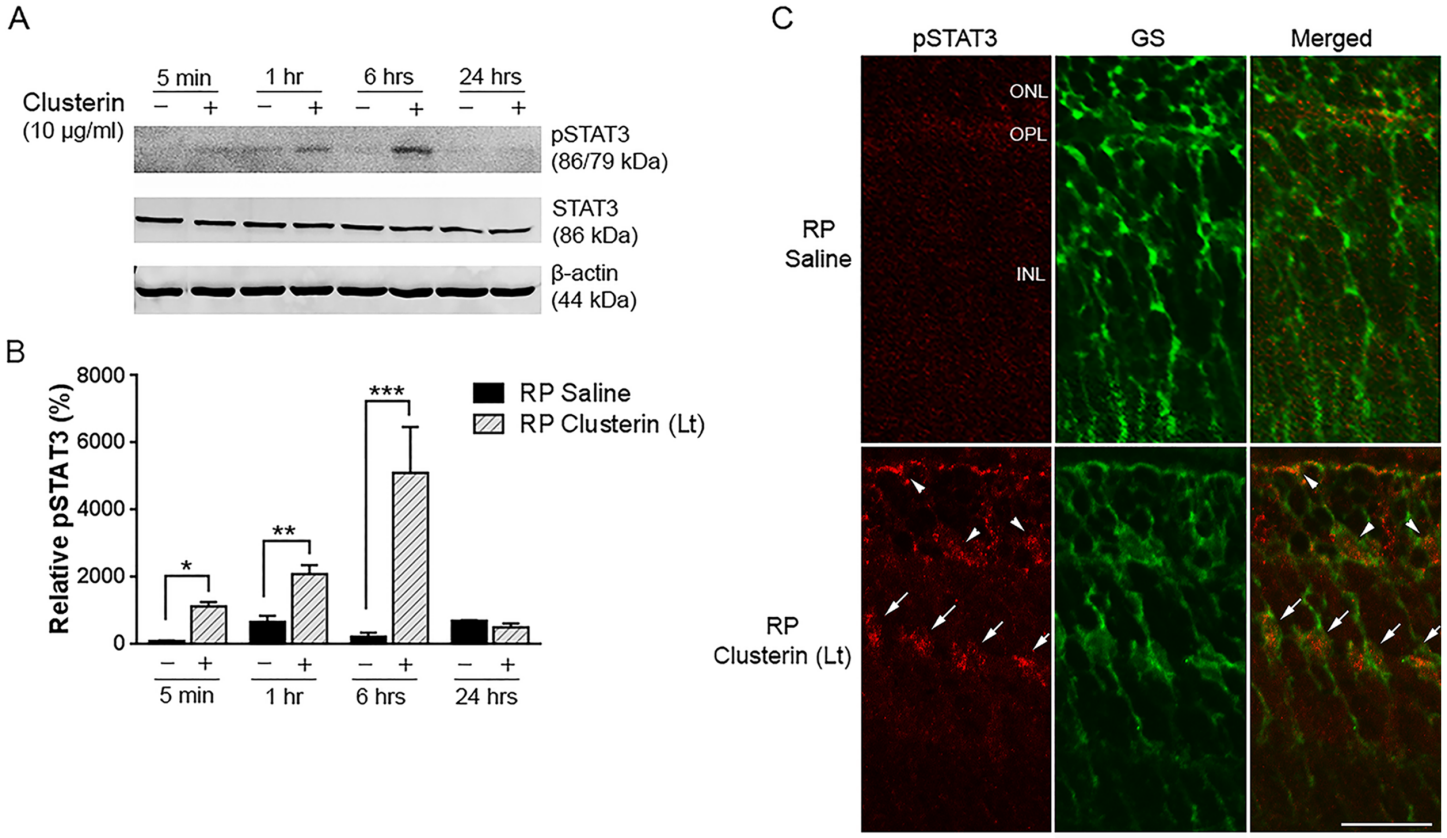
Up-regulation of STAT3 by clusterin in RP retina. The phosphorylated STAT3 immunoreactive protein was examined by immunoblot analysis in saline- and clusterin-treated RP retinas (A). Retinas were collected at 5 min, 1 hour, 6 hours, and 24 hours after injection at P15. Expression of pSTAT3 was up-regulated at 5 min. and peaked at 6hrs after injection of clusterin (+). In contrast, saline-treated retinas (-) showed slight activation of STAT3 throughout the time points after injection. Densitometry analysis of pSTAT3 was shown by measuring the intensity relative to control β-actin (B). Beta-actin and STAT3 served as the loading control. Confocal micrographs of immunoreactivity of pSTAT3 (red) and GS (green) in vertical sections of P15 RP retina, which were collected 6 hours after saline and clusterin injection (C). In saline-treated RP retina, no pSTAT3 immunoreactivity was observed. In clusterin-treated RP retina, pSTAT3 immunoreactivity was detected in the processes in the ONL and INL. Double-labeling experiments showed that processes of Müller cells and their cell bodies were co-localized with pSTAT3 immunoreactivity (arrows). In addition, some processes of Müller cells in the ONL were also labeled with pSTAT3 (arrowheads). Bars mean ± SEM, *P = 0.0224, **P = 0.0031, *** P<0.0001 ONL: outer nuclear layer; OPL: outer plexiform layer; INL: inner nuclear layer. STAT3, Signal Transducer and Activator of Transcription 3; pSTAT3, Phosphorylated Signal Transducer and Activator of Transcription 3; GS, Glutamate Synthase, Scale bar, 50 μm.

Previous studies have shown that activation of STAT3 pathways in Müller cells promotes photoreceptor survival in retinal degeneration [72]. We further investigated in this RP model the impact of clusterin on the STAT3 pathway by examining the phosphorylation of STAT3 in the retina after clusterin injection by immunoblot analysis. STAT3 phosphorylation was gradually increased from 5 minutes and peaked at 6 hours after clusterin treatment (Fig 7A). In contrast, in the saline treated group, pSTAT3 expression was weak. Fig 7B showed quantitative analysis of phosphorylation of STAT3 that was performed by measuring the intensity relative to control. After clusterin application, the phosphorylation of STAT3 was significantly higher at 5 min, 1 hour, and 6 hours than that of saline treated retinas (*P = 0.0224, **P = 0.0031, ***P<0.0001). After 24 hours of post-clusterin injection, phosphorylation of STAT3 was decreased. Beta-actin and STAT3 were shown as loading controls.

To localize clusterin-induced STAT3 phosphorylation, eyes were collected 6 hours after saline and clusterin injection. In saline-treated RP retina, no phosphorylated STAT3 immunoreactivity was observed. In clusterin-treated RP retina, phosphorylated STAT3 immunoreactivity was in the outer nuclear layer (Fig 7C, arrowheads) and some cells in the INL of the retina (Fig 7C, arrows). Immunohistochemical dual labeling experiments using antisera against both pSTAT3 and GS showed that some processes of Müller cells in the ONL and their cell bodies in the INL were co-localized with pSTAT3 immunoreactivity (Fig 7C). These results suggest that clusterin induced STAT3 activation in Müller cells.

### Clusterin affects survival of the rod photoreceptor cells via Bax suppression

**Fig 8:**
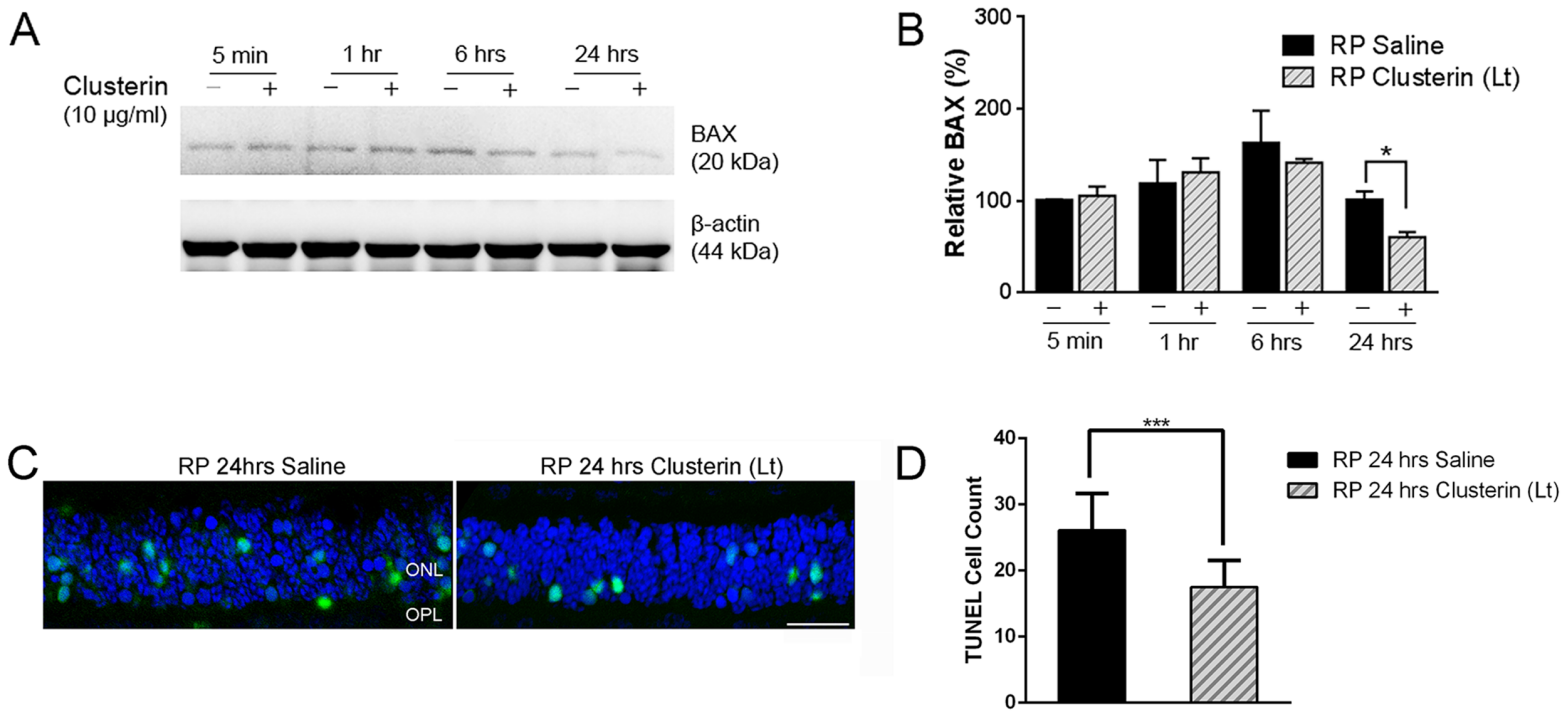
Suppression of Bax by clusterin in RP retina. Bax expression level was evaluated by immunoblot analysis in saline- and clusterin-treated RP retinas (A). Retinas were collected at 5 min, 1 hour, 6 hours, and 24 hours after injection at P15. Bax expression was significantly decreased at 24 hours after clusterin injection (+) compared to 24 hours of saline injection (-). Densitometry analysis of Bax expression was shown by measuring the intensity relative to the control β-actin (B). Data represents mean ± SEM, * P = 0.0098. Confocal micrographs taken from vertical cryostat sections (10μm-thick) processed for TOPRO-3 staining (blue) and TUNEL staining (green) in the 24 hour saline-treated and 24 hour clusterin-treated P15 retinas. TUNEL positive cells were more visible in the saline-treated retinas compared to clusterin-treated retinas (C). Histogram showing the number of TUNEL positive cells in the different comparisons (i.e. RP 24hrs saline vs. RP 24hrs Clusterin (Lt), n = 5). Counts refer to TUNEL positive cells encountered within a linear extension of 150 μm at the mid-peripheral region of the retina. Data represents mean ± SEM,*** P = 0.0009.

Several studies have reported that clusterin played a role in anti-apoptosis by suppressing Bax in specific forms of cancer [80, 81]. It was also seen in the S334ter model that there was a higher expression of Bax than other RP models such as *rd1*, *rd2*, *rd10*, *P23H*[82]. Taken together, we examined Bax expression in the retina after clusterin treatment at P15 by immunoblot analysis. A significant decrease in Bax expression was detected at 24 hours after clusterin injection, compared to 24 hours of saline injection (Fig 8A). Quantitative analysis was performed by measuring the intensity relative to control (Fig 8B). The expression of Bax was down-regulated by approximately 40% at 24 hours after clusterin treatment (*P = 0.0098) than saline treated retinas. We also observed down-regulation of cleaved caspase-3 by clusterin in RP retina (S3 Fig), showing the corresponding result of Bax down-regulation by clusterin at 24 hours, supporting that Bax-dependent up-regulation of cleaved caspase-3 was the main determinant of apoptosis in neurons [83]. Beta-actin was shown as a loading control to obtain relative Bax and cleaved caspase-3 (Fig 8, S3 Fig). Furthermore, intravitreal injection of clusterin at P15 resulted in significant reduction of TUNEL positive cells in ONL when examined at 24 hours after clusterin injection compared to 24 hours after saline injection (Fig 8C). Analysis of sections stained with TUNEL revealed approximately 26.1 ± 1.8 and 17.5 ± 1.3 TUNEL positive cells per 150 μm field in the 24 hour saline-treated and 24 hour clusterin-treated P15 retinas, respectively (Fig 8D). These results indicated that clusterin played a role in rod survival in RP retina through Bax suppression.

## Discussion

### Clusterin expression is enhanced in S334ter RP rat retina

Clusterin is a well-known factor up-regulated in many diseases; however, as an enigmatic chaperone protein with multiple actions, it may have previously unappreciated attributes that will contribute to an effective treatment to slow photoreceptor loss and prevent visual loss.

We observed enhanced expression of the clusterin precursor in the RP retinas compared to normal retinas (Fig 1A and 1B). However, no significant differences in the expression levels of clusterin-α were detected between the two groups (Fig 1A, S1 Fig, S2 Table). A possible explanation for the inconsistent expression of the precursor and clusterin-α is incomplete cleavage into a heterodimer complex of α and β subunits due to oxidative stress, or alternatively, an imbalance of potassium levels inducing stress-related genes [84–87]. Although the precise mechanism that leads to this increased clusterin expression in RP retinas is currently unresolved, previous studies have shown compensatory properties of clusterin to defend against oxidative stress, complement or chronic inflammation in other pathophysiologic conditions involving the exocrine pancreas, pulmonary vasculature and renal glomerulus [88–90]. Furthermore, a potential role to protect neurons from glutamate toxicity is known to induce clusterin expression in glial cells [38, 91]. Müller cells have a high-affinity glutamate carrier and convert glutamate to glutamine to clear the excess of glutamate after neuronal damage [92, 93]. Similarly, in RP, levels of excessive free glutamate are observed in the retina [94]. Thus, in the RP retina, hyper-reactive gliosis is common because the Müller glial cell is responding to excessive glutamate levels [11]. Therefore, we suggest that up-regulation of clusterin expression in Müller cells is due, in part, to glutamate toxicity in S334ter and helps maintain retinal cell viability.

### Clusterin effects on both eyes (bilateral effects) of the RP animal

We found that clusterin has a bilateral effect to rod cell survival and rod cell distribution (Fig 3). Furthermore, clusterin level changed in both control eye and clusterin injected eye of the same animal (Fig 4). What could be the possible mechanisms of a bilateral effect with clusterin treatment? We suggest that clusterin intravitreal treatment in one eye passes through the blood-retinal-barrier and acts on the saline-treated retina. In fact, evidence exists that clusterin may regulate the vascular smooth muscle cells, which in turn, may affect the blood-retinal barrier [55, 95, 96]. Previous reports suggest that clusterin has a permeability effect on the blood-brain-barrier in patients with Alzheimer’s disease (AD) [36, 86, 97]. Furthermore, bilateral effects in both eyes have been shown for specific reagents being used for ophthalmic treatment, including bevacizumab, a targeted anti-VEGF drug that has known effects on vascular permeability and revealed a significant decrease in the macular thickness of both eyes [98, 99]. Thus, clusterin treatment may have the ability to modify the vasculature of the blood-retinal-barrier, to penetrate systemically, and to affect the saline-treated contralateral eye of RP (Figs 3 and 4).

### Clusterin has impact on distribution of rods in RP retina

In S334ter-line3 RP retina, rods die in clusters and forms holes in the rod mosaic [21]. In this study, we showed that the distribution of rod cells was more homogenous in clusterin-treated retina at P30 (Fig 3). We also found that clusterin delays rod death (Figs 3 and 5). Thus, we suggest that clusterin may compensate a local deficit of survival factors caused by RP mutation [100] and decrease the probability of its neighbors to die (i.e. preventing cluster-form of rod death and hole-formation in rod mosaic). Alternatively, clusterin affects the extracellular matrix (ECM) through metalloproteinase matrix protein-9 (MMP-9) in RP retina to rearrange rods. This hypothesis is supported by published data that SB-3CT, a specific inhibitor of MMP-9 and MMP-2, is involved in modulating the cone distribution in RP retina through ECM [22, 59, 60]. Additionally, it has been reported that clusterin interacts with MMP-9 to reorganize the tissue by modulating ECM in corneal epithelial cells of the dry eye [101] and in immune cells such as monocytes and macrophages in vitro [102].

### Clusterin has a protective effect in RP retinas

There are three isoforms of clusterin: (1) nuclear, (2) secretory, and (3) cytoplasmic. The nuclear clusterin binds Ku70 to release Bax and induces apoptosis, whereas the cytoplasmic and secretory clusterin inhibit apoptosis [103]. The secretory form of clusterin is synthesized as a ~60kDa protein precursor and becomes heavily glycosylated and cleaved into an alpha and beta chain, held together by disulfide bonds in the mature secreted heterodimer protein form (~70kDa) [104, 105]. We observed a significant increase in clusterin in both precursor-clusterin (~70kDa) and clusterin-α (36-39kDa) as early as 5 minutes and its enhanced expression continues to increase until 48 hours in both RP Clusterin (Lt) and RP Saline (Rt) compared to that of RP saline retinas (Fig 4). These results clearly suggest that the secretion pathway is facilitated kinetically relative to the cleavage pathway. In addition, up-regulation of both precursor-clusterin and clusterin-α after clusterin injection implies that more endogenous precursor clusterin is produced in the retina and protected the rods (Fig 5). Our findings have potential therapeutic implications, as these data suggest that single or multiple treatments with clusterin improve rod survival (Fig 5). In addition, we hypothesize that clusterin’s beneficial effect on rod survival is not driven by rods dying in clusters. In P30 RP saline, we observed rod holes (Fig 3A). However, at P30 the rods were homogenously distributed throughout the retina after injection of clusterin at P15 (Fig 3E). When we inject clusterin at P30 again (i.e. retinas with homogenously distributed rods) and quantify the rod cells at P45, we observed more rod survival than that of single treatment of clusterin. Therefore, our result demonstrates that clusterin has effects on rod survival whether or not rod holes are present. The existence of dark patches or ‘‘holes of photoreceptors” in cyclin D1 (cd1) mutant and P23H-line1 rat has been reported [106, 107]. In addition, human retinal dystrophy, inherited retinal degeneration, and photo-pigment genetic perturbations in M-opsin cones showed a similar pattern in ONL [108–111]. We believe that clusterin treatment will work on photoreceptor survival in these and other degenerative retinas regardless of holes or rings. In the future, we will analyze potential survival effects of clusterin in long-term by developing clusterin-secreting microdevices and implanting them intravitreally into the eyes of RP animals. In addition, we will also investigate the visual function to examine the retinal physiology of photoreceptor/retinal ganglion cells [23] at different times after clusterin application.

What are the possible mechanisms underlying rod survival with the secretory form of clusterin? In our study, we did not observe clusterin expression in photoreceptor cells in RP retinas (Fig 1) whereas previous papers showed clusterin expression in retinal neurons. The discrepancies between previous studies and our study may be due to different immunological staining protocols or using alternative clusterin antibodies. The up-regulation of clusterin was reported in the animal models of retinal degeneration such as rds/rds mouse [42] and light-induced damage [43, 44, 46, 48, 49, 104] in which photoreceptor death occurs by apoptosis. Oxidative stress in RP retina leads to photoreceptor death. The production of reactive oxygen species causes oxidative damage to proteins, lipids, and DNA [112, 113]. Furthermore, outer segment membranes of rods are lipid rich and susceptible to oxidation [114]. Although we did not observe clusterin expression in the photoreceptors in the RP retina, the application of clusterin may minimize the effects of peroxidized membrane lipids [115]. This hypothesis is supported by the presence of clusterin in the inner segments of the dying photoreceptors in degenerative retinas [47]. In addition, oxidative metabolism occurs in the mitochondria in the inner segment of the photoreceptors [116]. Furthermore, there is evidence that clusterin localized to mitochondria and inhibited apoptosis by interacting with activated Bax [80].

In S334ter RP retina, Bax is highly expressed in the early stage of retinal development [82]. Bax plays a role in mediating of apoptosis via the caspase cascade in outer membrane of mitochondria [117–119]. The mitochondrial function as an energy reservoir is important to maintain photoreceptors against retinal degeneration [120–122]. Thus, increased expression of Bax in RP retina and its down-regulation by clusterin treatment suggests that clusterin, may in part, protect rod photoreceptors by suppressing the crucial mitochondrial pathway of the cell death (Fig 8). Our results also showed that intravitreal treatment with clusterin reduces TUNEL positive cells after 24hours of clusterin post-injection (Fig 8C and 8D). The results indicate that down-regulation of Bax at 24 hours with clusterin may lead to reduction of cell death. Furthermore, intravitreal injection of clusterin at P15 resulted in significant protection of ONL when examined at P21 and P30. Our results clearly demonstrated that inhibition of Bax after 24 hours of clusterin post- injection was significant enough to cause effects on the number of rods at the later stage of S334ter retinas (Figs 3 and 5). Further support for the suggestion comes from clusterin inhibiting mitochondrial apoptosis through up-regulation of Akt phosphorylation in other systems [51, 80, 81, 123]. Thus, activation of Akt and suppression of Bax with clusterin may contribute, in part, to protect rod photoreceptors in a later stage of S334ter retinas. In support of these findings, photoreceptor cell death in the rd1 mouse retina is associated with inactivation of the Akt survival pathway [118]. Future in vivo studies will address the specific mechanisms by which clusterin promotes survival of rods.

In RP retina, we also observed up-regulation of pSTAT3 in Müller cells after clusterin treatment (Fig 7). In the past, efforts to rescue photoreceptors through manipulation of the STAT3 pathway in vitro or in vivo have produced encouraging results in animal models of retinal degeneration [124, 125]. Activation of the STAT3 pathway in Müller cells by trophic factors such as IL-6 family members—including Oncostatin M (OSM), leukemia inhibitory factor (LIF), cardiotrophin 1 (CT-1), and ciliary neurotrophic factor (CNTF)—regulates the phototransduction apparatus in photoreceptor cells and lead to rescue of photoreceptors [72, 125–130]. In addition, activation of STAT3 and inhibition of Bax activity via clusterin has been documented in hypothalamus, plasma, and cancer cells [131–133]. Thus, our data suggest that exogenous application of clusterin in RP activates STAT3 in Müller cells that may promote survival signaling and protect the rod photoreceptors. Müller cells produce protective factors including IL-6 and leptin in response to stress-induced retinal degeneration and regulate photoreceptor protection [128, 130, 134]. Additionally, IL-6 and leptin cytokines have a synergistic effect on retinal regeneration by stimulating STAT3 activation in Müller cells [134]. Thus, clusterin which is known to have role in modulating IL-6/STAT3 activation by suppressing Bax in cancer cells [131], enhancing leptin-induced STAT3 activation in hypothalamus [132] and regulating leptin activity in plasma [133], may trigger the IL-6 and leptin pathways to allow survival of photoreceptors in RP via STAT3 activation on Müller cells. There is also evidence that clusterin receptors are expressed in photoreceptors and Müller cells. Identified clusterin receptors are the Transforming Growth Factor (TGF) beta receptor type I and II [135] and clusterin binds with high affinity to the multi-ligand receptors apolipoprotein E receptor 2 (ApoER2) and very low-density lipoprotein receptor (VLDLR) [136]. The VLDLR is present in the photoreceptors [137]. In addition, TGF-beta 1 and—beta 2 have been found in the photoreceptor inner and outer segments, RPE cells, Müller cells, ganglion cells, and vascular-associated cells [138–140]. Thus, clusterin’s cell survival action may be through these receptors [141, 142]. Alternatively, clusterin may act as heat-shock proteins in the oxidative-stress condition because clusterin and small heat-shock proteins are molecular chaperones that share many functional similarities [52, 143].

## Conclusion

In this study, we investigated if an exogenous application of clusterin could affect the rod survival in RP retina, with the hypothesis that clusterin was an “innate defender.” Our data showed neuroprotective features of clusterin in a transgenic rat model of RP. Our study clearly demonstrated that clusterin treatment promotes rod cell survival by activating both Akt and STAT3 while deactivating Bax. These effects are encouraging and so dramatic that clusterin may be a potential therapeutic agent in slowing the progression of vision loss in human RP.

## Supporting information

S1 Fig Densitometry analysis of clusterin-α bands. Densitometry analysis of clusterin-α bands at P15, P30, and P60 in normal and RP retinas was shown by measuring the intensity relative to the control β-actin. Data represents mean ± SEM.
(TIF)

S2 Fig TOPRO-3 staining in RP Saline and RP Clusterin (Lt) retinas. Confocal micrographs taken from vertical cryostat sections processed for TOPRO-3 staining in P21-, P30-, P45-RP Saline and P21-, P30-, P45-RP Clusterin (Lt) retinas. The thickness of the ONL for P21, P30, and P45 RP Saline retinas was 13.2 ± 0.2 μm, 10 ± 0.2 μm and 4.2 ± 0.15 μm, respectively. The thickness of the ONL for P21, P30, and P45 RP Clusterin (Lt) retinas was 14.5 ± 0.3 μm, 12 ± 0.3 μm and 4.3 ± 0.1 μm, respectively. ONL, outer nuclear layer; OPL, outer plexiform layer; INL, inner nuclear layer; IPL, inner plexiform layer; GCL, ganglion cell layer; P, postnatal; N, normal; RP, Retinitis Pigmentosa. Data represents mean ± SEM. Scale bar = 20 μm. Data represents mean ± SEM, *** P<0.001.
(TIF)

S3 Fig Expression of Cleaved caspase- 3 in saline- and clusterin-treated RP retinas. Cleaved caspase-3 expression level was evaluated by immunoblot analysis in saline- and clusterin-treated RP retinas (A). Retinas were collected at 5 min, 1 hour, 6 hours, and 24 hours after injection at P15. Cleaved caspase-3 expression was significantly decreased at 24 hours after clusterin injection (+) compared to 24 hours of saline injection (-). Densitometry analysis of cleaved caspase-3 expression was shown by measuring the intensity relative to the control β-actin (B). Data represents mean ± SEM, *** P<0.001.
(TIF)

S1 Table Quantification of clusterin precursor expression in normal vs RP retinas by immunoblot analysis. Legend: Intensity of immunoreactive bands of clusterin precursor in RP retinas compared to normal retinas.
(DOCX)

S2 Table Quantification of clusterin-α expression in normal vs RP retinas by immunoblot analysis. Legend: Intensity of immunoreactive bands of clusterin- α in RP retinas compared to normal retinas.
(DOCX)

S3 Table Quantification of rhodopsin-immunoreactive rods in RP Saline, RP Saline (Rt) and RP Clusterin (Lt) P30 retinas. Legend: The rhodopsin-immunoreactive rods were counted from the 1 x 1 mm^2^ sampling areas of whole-mount retinas (Fig 3H).
(DOCX)

S4 Table The coefficient of clustering of rods in RP Saline, RP Saline (Rt) and RP Clusterin (Lt) P30 S334ter retinas. Legend: The coefficient of clustering was measured in all groups (Fig 3I).
(DOCX)

S5 Table Quantification of clusterin precursor and clusterin-α expression in RP Saline (Control), RP Saline (Rt), and RP Clusterin (Lt) retinas by immunoblot analysis. Legend: Immunoblot analysis shows up-regulation of clusterin precursor and clusterin-α in both RP Saline (Rt) and RP Clusterin (Lt) retinas compared to RP Saline retinas. Beta actin was used as loading control to obtain relative clusterin precursor (Fig 4B) and clusterin-α expression (Fig 4C).
(DOCX)

S6 Table Quantification of rhodopsin-immunoreactive rods in RP saline, RP Clusterin Single (Lt), and RP Clusterin Multiple (Lt) retinas. Legend: The rhodopsin-immunoreactive rods were counted from the 1 x 1 mm^2^ sampling areas of whole-mount retinas (Fig 5B).
(DOCX)

S7 Table Quantification of pAKT expression in RP Saline vs RP Clusterin (Lt) retinas by immunoblot analysis. Legend: Immunoblot analysis shows up-regulation of pAKT expression in RP Clusterin (Lt) retina compared to RP Saline retinas from 5 minutes after injection at P15. Beta actin was used as loading control to obtain relative pAKT expression (Fig 6B).
(DOCX)

S8 Table Quantification of pSTAT3 expression in RP Saline vs RP Clusterin (Lt) retinas by immunoblot analysis. Legend: Immunoblot analysis shows up-regulation of pSTAT3 expression in RP Clusterin (Lt) retina compared to RP Saline retinas at 5minutes, 1 hour, and 6 hours after injection at P15. Beta actin was used as loading control to obtain relative pSTAT3 expression (Fig 7B).
(DOCX)

S9 Table Quantification of BAX expression in RP Saline vs RP Clusterin (Lt) retinas by immunoblot analysis. Legend: Immunoblot analysis shows suppression of BAX at 24hours after clusterin injection at P15. Beta actin was used as loading control to obtain relative BAX expression (Fig 8B).
(DOCX)
