# Supplementary figures and images for: Protective effect of clusterin on rod photoreceptor in rat model of retinitis pigmentosa
Source: PLoS One. 2017 Aug 2;12(8):e0182389. doi: 10.1371/journal.pone.0182389 (PMC5540409; doi:10.1371/journal.pone.0182389)

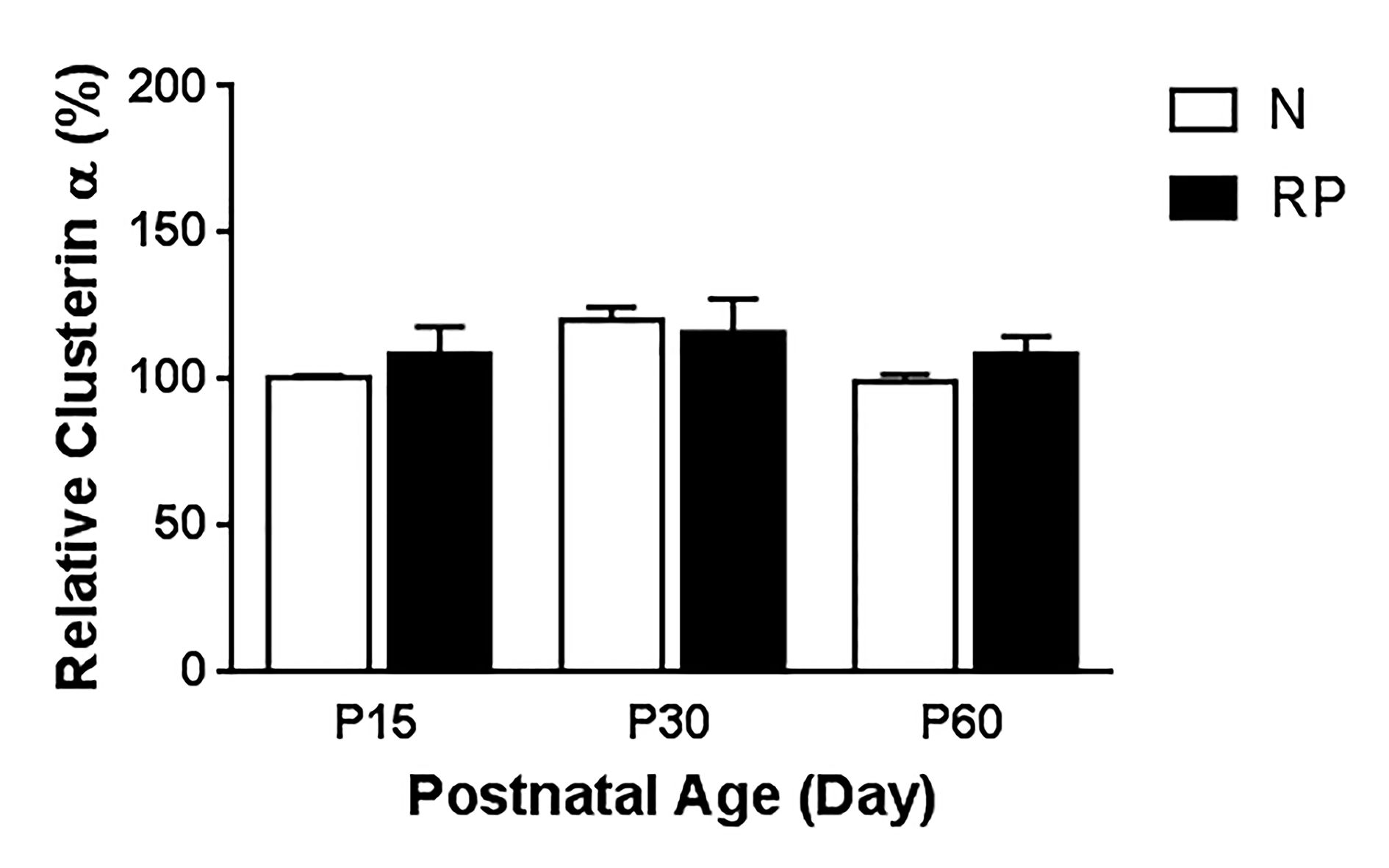

Supplement: S1 Fig — Densitometry analysis of clusterin-α bands at P15, P30, and P60 in normal and RP retinas was shown by measuring the intensity relative to the control β-actin. Data represents mean ± SEM. (TIF) [file pone.0182389.s001.tif]

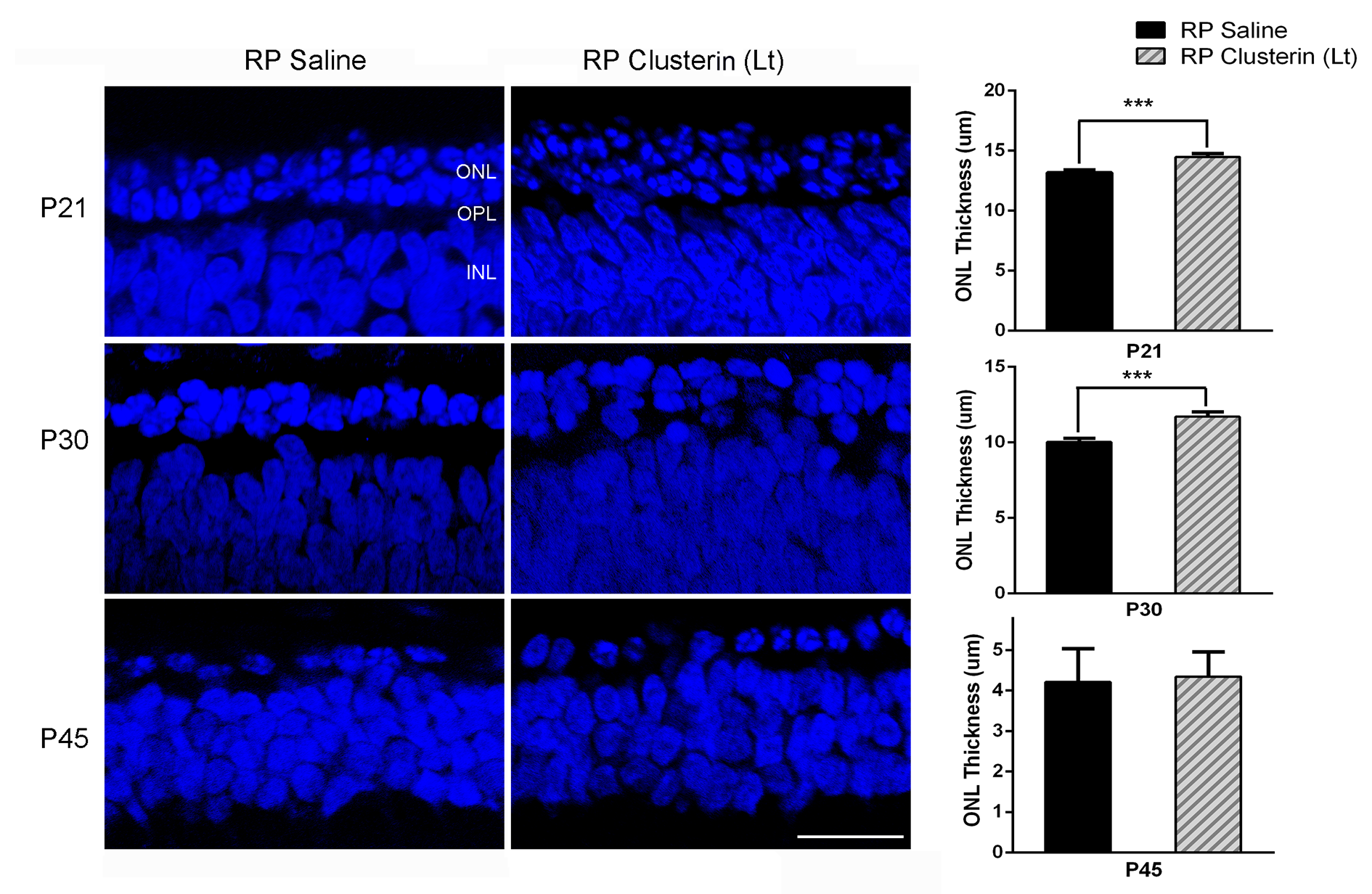

Supplement: S2 Fig — Confocal micrographs taken from vertical cryostat sections processed for TOPRO-3 staining in P21-, P30-, P45-RP Saline and P21-, P30-, P45-RP Clusterin (Lt) retinas. The thickness of the ONL for P21, P30, and P45 RP Saline retinas was 13.2 ± 0.2 μm, 10 ± 0.2 μm and 4.2 ± 0.15 μm, respectively. The thickness of the ONL for P21, P30, and P45 RP Clusterin (Lt) retinas was 14.5 ± 0.3 μm, 12 ± 0.3 μm and 4.3 ± 0.1 μm, respectively. ONL, outer nuclear layer; OPL, outer plexiform layer; INL, inner nuclear layer; IPL, inner plexiform layer; GCL, ganglion cell layer; P, postnatal; N, normal; RP, Retinitis Pigmentosa. Data represents mean ± SEM. Scale bar = 20 μm. Data represents mean ± SEM, *** P<0.001. (TIF) [file pone.0182389.s002.tif]

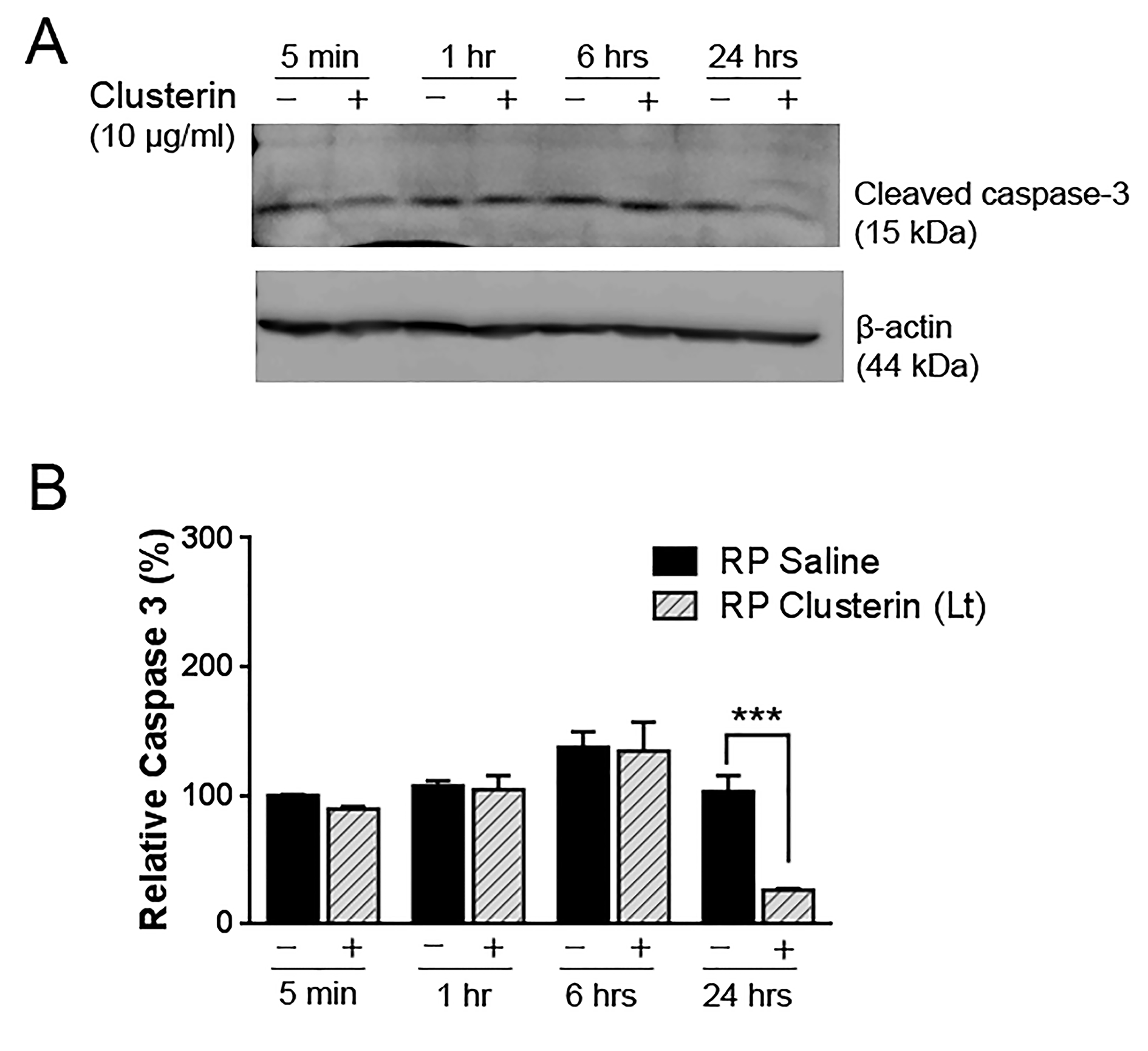

Supplement: S3 Fig — Cleaved caspase-3 expression level was evaluated by immunoblot analysis in saline- and clusterin-treated RP retinas (A). Retinas were collected at 5 min, 1 hour, 6 hours, and 24 hours after injection at P15. Cleaved caspase-3 expression was significantly decreased at 24 hours after clusterin injection (+) compared to 24 hours of saline injection (-). Densitometry analysis of cleaved caspase-3 expression was shown by measuring the intensity relative to the control β-actin (B). Data represents mean ± SEM, *** P<0.001. (TIF) [file pone.0182389.s003.tif]
