# Supplementary material for: Protective effect of clusterin on rod photoreceptor in rat model of retinitis pigmentosa
Source: PLoS One. 2017 Aug 2;12(8):e0182389. doi: 10.1371/journal.pone.0182389 (PMC5540409; doi:10.1371/journal.pone.0182389)
Supplement: S1 Table — Legend: Intensity of immunoreactive bands of clusterin precursor in RP retinas compared to normal retinas. (DOCX) [file pone.0182389.s004.docx]

|  | Normal **(clusterin precursor)** | | | RP (**clusterin precursor)** | | |
| --- | --- | --- | --- | --- | --- | --- |
| P15 | 100.4647 | 100.0175 | 100.2965 | 337.9715 | 333.4964 | 397.7004 |
| P30 | 113.2347 | 105.7657 | 131.4690 | 327.8545 | 346.1868 | 440.9697 |
| P60 | 116.1272 | 122.8685 | 139.6454 | 289.1152 | 330.3028 | 371.5251 |

**S1 Table. Quantification of clusterin precursor expression in normal vs RP retinas by immunoblot analysis.**
