# Supplementary material for: Protective effect of clusterin on rod photoreceptor in rat model of retinitis pigmentosa
Source: PLoS One. 2017 Aug 2;12(8):e0182389. doi: 10.1371/journal.pone.0182389 (PMC5540409; doi:10.1371/journal.pone.0182389)
Supplement: S2 Table — Legend: Intensity of immunoreactive bands of clusterin- α in RP retinas compared to normal retinas. (DOCX) [file pone.0182389.s005.docx]

**S2 Table. Quantification of clusterin-α expression in normal vs RP retinas by immunoblot analysis.**

|  | Normal ( **clusterin-α** ) | | | RP ( **clusterin-α** ) | | |
| --- | --- | --- | --- | --- | --- | --- |
| P15 | 101.0760 | 100.0586 | 100.9663 | 98.29957 | 115.6412 | 112.4999 |
| P30 | 115.1061 | 124.4777 | 120.9594 | 104.6803 | 115.9779 | 127.6023 |
| P60 | 101.1830 | 96.35850 | 100.0601 | 106.8365 | 115.5461 | 103.7477 |
