# Supplementary material for: Protective effect of clusterin on rod photoreceptor in rat model of retinitis pigmentosa
Source: PLoS One. 2017 Aug 2;12(8):e0182389. doi: 10.1371/journal.pone.0182389 (PMC5540409; doi:10.1371/journal.pone.0182389)
Supplement: S3 Table — Legend: The rhodopsin-immunoreactive rods were counted from the 1 x 1 mm2 sampling areas of whole-mount retinas (Fig 3H). (DOCX) [file pone.0182389.s006.docx]

**S3 Table. Quantification of rhodopsin-immunoreactive rods in RP Saline, RP Saline (Rt) and RP Clusterin (Lt) P30 retinas.**

| **P30** | **RP (Saline)** | **RP Saline (Rt)** | **RP Clusterin (Lt)** |
| --- | --- | --- | --- |
| Animal 1 | 3472 | 9486 | 10616 |
| Animal 2 | 4190 | 6242 | 9563 |
| Animal 3 | 4649 | 6861 | 8772 |
