# Supplementary material for: Protective effect of clusterin on rod photoreceptor in rat model of retinitis pigmentosa
Source: PLoS One. 2017 Aug 2;12(8):e0182389. doi: 10.1371/journal.pone.0182389 (PMC5540409; doi:10.1371/journal.pone.0182389)
Supplement: S4 Table — Legend: The coefficient of clustering was measured in all groups (Fig 3I). (DOCX) [file pone.0182389.s007.docx]

|  | RP Saline | | | RP Saline (Rt) | | | RP Clusterin (Lt) | | |
| --- | --- | --- | --- | --- | --- | --- | --- | --- | --- |
| CC | 2.0053 | 1.9156 | 1.876367 | 1.6356 | 1.5494 | 1.5439 | 1.4332 | 1.2849 | 1.4822 |

**S4 Table. The coefficient of clustering of rods in RP Saline, RP Saline (Rt) and RP Clusterin (Lt) P30 S334ter retinas.**
