# Supplementary material for: Protective effect of clusterin on rod photoreceptor in rat model of retinitis pigmentosa
Source: PLoS One. 2017 Aug 2;12(8):e0182389. doi: 10.1371/journal.pone.0182389 (PMC5540409; doi:10.1371/journal.pone.0182389)
Supplement: S5 Table — Legend: Immunoblot analysis shows up-regulation of clusterin precursor and clusterin-α in both RP Saline (Rt) and RP Clusterin (Lt) retinas compared to RP Saline retinas. Beta actin was used as loading control to obtain relative clusterin precursor (Fig 4B) and clusterin-α expression (Fig 4C). (DOCX) [file pone.0182389.s008.docx]

**S5 Table. Quantification of clusterin precursor and clusterin-α expression in RP Saline (Control), RP Saline (Rt), and RP Clusterin (Lt) retinas by immunoblot analysis.**

Clusterin Precursor

|  | RP Clusterin (Lt) | | | RP Saline (Rt) | | |
| --- | --- | --- | --- | --- | --- | --- |
| Control | 100.3543 | 99.4125 | 100.9820 | 100.2436 | 100.9723 | 100.5134 |
| 5 min | 483.7518 | 497.9798 | 533.5498 | 432.475 | 445.1948 | 476.9944 |
| 30 min | 383.8169 | 395.1056 | 423.3275 | 439.3963 | 452.3197 | 484.6283 |
| 1 hr | 755.5445 | 777.7663 | 833.3211 | 544.1926 | 566.0807 | 620.8007 |
| 6 hrs | 799.1973 | 822.7031 | 881.4676 | 599.8753 | 617.5186 | 661.6271 |
| 24 hrs | 906.6968 | 933.3643 | 1000.033 | 750.7210 | 775.7422 | 838.2953 |
| 48 hrs | 1561.233 | 1607.1510 | 1721.9480 | 1297.4910 | 1335.6520 | 1431.0560 |

Clusterin alpha

|  | RP Clusterin (Lt) | | | RP Saline (Rt) | | |
| --- | --- | --- | --- | --- | --- | --- |
| Control | 102.69400 | 99.31890 | 105.39110 | 100.39040 | 100.39130 | 100.53970 |
| 5 min | 538.8095 | 553.5714 | 590.4762 | 491.0006 | 504.4527 | 538.0829 |
| 30 min | 423.2840 | 429.4014 | 444.6948 | 469.2566 | 482.1129 | 514.2538 |
| 1 hr | 766.5805 | 787.5827 | 840.0882 | 572.7065 | 593.8765 | 646.8016 |
| 6 hrs | 847.7966 | 871.0239 | 929.0922 | 666.0298 | 687.0169 | 739.4847 |
| 24 hrs | 1246.2070 | 1280.3490 | 1365.7060 | 942.7377 | 968.5662 | 1033.1370 |
| 48 hrs | 1771.9070 | 1820.4520 | 1941.8150 | 1076.4100 | 1105.9010 | 1179.6270 |
