# Supplementary material for: Protective effect of clusterin on rod photoreceptor in rat model of retinitis pigmentosa
Source: PLoS One. 2017 Aug 2;12(8):e0182389. doi: 10.1371/journal.pone.0182389 (PMC5540409; doi:10.1371/journal.pone.0182389)
Supplement: S6 Table — Legend: The rhodopsin-immunoreactive rods were counted from the 1 x 1 mm2 sampling areas of whole-mount retinas (Fig 5B). (DOCX) [file pone.0182389.s009.docx]

|  | RP Clusterin Repeated (Lt) | | | RP Clusterin Single (Lt) | | | RP Saline | | |
| --- | --- | --- | --- | --- | --- | --- | --- | --- | --- |
| P45 | 9408 | 8248 | 8714 | 7440 | 6314 | 7530 | 542 | 500 | 459 |
| P60 | 4048 | 3968 | 3948 | 1120 | 1242 | 1203 | 16 | 18 | 21 |
| P75 | 1612 | 2620 | 2312 | 504 | 884 | 744 | 5 | 7 | 8 |

**S6 Table. Quantification of rhodopsin-immunoreative rods in RP saline, RP Clusterin Single (Lt), and RP Clusterin Multiple (Lt) retinas.**
