# Supplementary material for: Protective effect of clusterin on rod photoreceptor in rat model of retinitis pigmentosa
Source: PLoS One. 2017 Aug 2;12(8):e0182389. doi: 10.1371/journal.pone.0182389 (PMC5540409; doi:10.1371/journal.pone.0182389)
Supplement: S7 Table — Legend: Immunoblot analysis shows up-regulation of pAKT expression in RP Clusterin (Lt) retina compared to RP Saline retinas from 5 minutes after injection at P15. Beta actin was used as loading control to obtain relative pAKT expression (Fig 6B). (DOCX) [file pone.0182389.s010.docx]

**S7 Table. Quantification of pAKT expression in RP Saline vs RP Clusterin (Lt) retinas by immunoblot analysis.**

|  | RP Saline | | | RP Clusterin (Lt) | | | |
| --- | --- | --- | --- | --- | --- | --- | --- |
| 5 min | 100.16620 | 100.74800 | 100.59850 | 709.22190 | 625.03330 | 649.38200 |  |
| 1 hr | 110.84410 | 122.56320 | 134.53510 | 512.01830 | 566.57710 | 523.44280 |  |
| 6 hrs | 30.40254 | 23.18608 | 33.35008 | 270.91470 | 319.83730 | 273.94070 |  |
| 24 hrs | 100.16620 | 100.74800 | 100.59850 | 271.93600 | 343.23200 | 315.40450 |  |
