# Supplementary material for: Protective effect of clusterin on rod photoreceptor in rat model of retinitis pigmentosa
Source: PLoS One. 2017 Aug 2;12(8):e0182389. doi: 10.1371/journal.pone.0182389 (PMC5540409; doi:10.1371/journal.pone.0182389)
Supplement: S8 Table — Legend: Immunoblot analysis shows up-regulation of pSTAT3 expression in RP Clusterin (Lt) retina compared to RP Saline retinas at 5minutes, 1 hour, and 6 hours after injection at P15. Beta actin was used as loading control to obtain relative pSTAT3 expression (Fig 7B). (DOCX) [file pone.0182389.s011.docx]

**S8 Table. Quantification of pSTAT3 expression in RP Saline vs RP Clusterin (Lt) retinas by immunoblot analysis.**

|  | RP Saline | | | RP Clusterin (Lt) | | | |
| --- | --- | --- | --- | --- | --- | --- | --- |
| 5 min | 100.55850 | 81.72762 | 125.41410 | 1061.62200 | 1056.51800 | 1286.25500 |  |
| 1 hr | 522.03840 | 862.08710 | 650.23220 | 2192.11100 | 2299.16800 | 1798.35800 |  |
| 6 hrs | 91.20378 | 287.64160 | 318.55420 | 6678.36000 | 4332.04300 | 4302.89100 |  |
| 24 hrs | 721.31390 | 705.24270 | 692.52460 | 576.94520 | 384.31140 | 577.02780 |  |
