# Supplementary material for: Protective effect of clusterin on rod photoreceptor in rat model of retinitis pigmentosa
Source: PLoS One. 2017 Aug 2;12(8):e0182389. doi: 10.1371/journal.pone.0182389 (PMC5540409; doi:10.1371/journal.pone.0182389)
Supplement: S9 Table — Legend: Immunoblot analysis shows suppression of BAX at 24hours after clusterin injection at P15. Beta actin was used as loading control to obtain relative BAX expression (Fig 8B). (DOCX) [file pone.0182389.s012.docx]

**S9 Table. Quantification of BAX expression in RP Saline vs RP Clusterin (Lt) retinas by immunoblot analysis.**

|  | RP Saline | | | RP Clusterin (Lt) | | |
| --- | --- | --- | --- | --- | --- | --- |
| 5 min | 100.54510 | 100.01910 | 102.11210 | 117.26820 | 98.30343 | 100.22860 |
| 1 hr | 148.30450 | 103.11980 | 105.51610 | 148.65630 | 119.81710 | 123.84700 |
| 6 hrs | 203.44730 | 140.38050 | 146.42220 | 144.84960 | 137.25590 | 142.99190 |
| 24 hrs | 110.86730 | 100.54160 | 92.78730 | 66.82065 | 58.33977 | 56.35509 |
